# Impact of climate warming on *Oncomelania hupensis* in China: multi-scale evidence

**DOI:** 10.1186/s40249-026-01475-0

**Published:** 2026-07-03

**Authors:** Qin Li, Jin-Xin Zheng, Li-Juan Zhang, Su-ying Guo, Ying Yang, Jürg Utzinger, Xiao-Nong Zhou, Jing Xu, Shi-Zhu Li

**Affiliations:** 1 National Institute of Parasitic Diseases at Chinese Center for Disease Control and Prevention (Chinese Center for Tropical Diseases Research), National Key Laboratory of Intelligent Tracking and Forecasting for Infectious Diseases, NHC Key Laboratory of Parasite and Vector Biology, WHO Collaborating Center for Tropical Diseases, National Center for International Research on Tropical Diseases, Ministry of Science and Technology, Shanghai, 200025 China; 2 School of Global Health, Chinese Center for Tropical Diseases Research, Shanghai Jiao Tong University School of Medicine, Shanghai, 200025 China; 3 Swiss Tropical and Public Health Institute, Kreuzstrasse 2, 4123 Allschwil, Switzerland

**Keywords:** *Oncomelania hupensis*, Climate warming, Land use, Temperature, Time window

## Abstract

**Background:**

Climate warming is increasingly reshaping the population dynamics of *Oncomelania hupensis*, the sole intermediate host of *Schistosoma japonicum* in China. Investigating the response of *O. hupensis* to climate warming is crucial for guiding targeted and precise interventions against these snails*,* thereby advancing the progress towards schistosomiasis elimination and sustaining the achievement of elimination in China. This study aims to quantify the multi-scale responses of *O. hupensis* to climate warming.

**Method:**

We conducted a 45-day temperature-controlled laboratory experiment with adult *O. hupensis* from Hubei Gongan County, China, exposing snails to the Low Temp group (5 ˚C), the Control group (25 ˚C), and the High Temp group (30 ˚C) to assess body-size-dependent survival. We then analyzed *O. hupensis* surveillance data from 1990 to 2022 across 12 provinces in China (34,554 villages), linked to climate data downloaded from ERA5-Land. We developed sliding-window models to examine climate exposure across life stages to assess population-level responses to climate warming, and used general linear mixed-effects models to explore the environmental factors influencing climate responses (geographic variables: latitude, longitude, altitude, land use, historical climate variability). Model performance was evaluated to identify the best predictive models, which were subsequently used to project future *O. hupensis* density under the SSP1-2.6, SSP2-4.5, and SSP5-8.5 scenarios. We further sampled 240 wild *O. hupensis* and measured their body sizes to validate the previous results inversely.

**Results:**

At the individual level, we observed that larger snails had a higher survival rate at both the Low Temp group (91.6%, 95% *CI* 85.8–97.7%) and High Temp group (45.7%, 95% *CI* 31.9–65.6%) compared with the control group (76.5%, 95% *CI* 58.7–99.5%). At the population level, *O. hupensis* density closely tracks climate warming, with 92.0% of populations showing positive temperature sensitivity. Exposure during the coldest (January: 2.7 × 10^–2^, 95% *CI* 3 × 10^–3^–5.1 × 10^–2^; February: 3.3 × 10^–2^, 95% *CI* 1.1 × 10^–2^–5.6 × 10^–2^) and breeding seasons (April: 2.4 × 10^–2^, 95% *CI* 6 × 10^–3^–4.3 × 10^–2^; September–October: 3.5 × 10^–2^, 95% *CI* 2 × 10^–3^–6.8 × 10^–2^) had a greater influence on *O. hupensis* density under climate warming. *O. hupensis* in waterbody-dominated areas showed the largest density growth rates (*ß* = 1.6 × 10^–1^, 95% *CI* 1.1 × 10^–1^–2.2 × 10^–1^), whereas crop populations were the most sensitive to land cover, a 1% increase in crop cover was associated with a 0.2 increase in population density. At the ecosystem level, only 19.0–24.6% of populations were expected to benefit from climate change by 2100, and these benefiting populations were concentrated in regions where sampled wild snails had a mean body size of 8.2 mm. Cropland will account for 72.0% of populations benefiting from climate warming, compared with 17.2% in forests and 8.3% in impervious-dominated areas.

**Conclusion:**

The *O. hupensis* populations grow under climate warming but are regulated by land-use types. To mitigate the impacts of climate warming and land-use on snails, surveillance and integrated interventions should be strengthened through multi-sector collaboration.

**Supplementary Information:**

The online version contains supplementary material available at 10.1186/s40249-026-01475-0.

## Background

Schistosomiasis is a vector-borne parasitic disease caused by blood flukes (trematode worms) of the genus S*chistosoma*. With more than 800 million people living in regions at risk of infection and over 240 million people infected, schistosomiasis ranks second after malaria in disease burden among parasitic diseases [1, 2]. In 2021, the World Health Organization (WHO) launched a strategic plan “Ending the neglect to attain the sustainable development goals: a sustainability framework for action against neglected tropical diseases 2021–2030” with an imperative goal for member states to eliminate schistosomiasis as a public health problem and interrupt transmission of schistosomiasis in humans in selected countries by 2030 [3]. However, there hasn’t been as much progress as expected [4, 5]. Given that schistosomes have recently been shown to overwinter by infecting adapted intermediate host snails and to produce viable offspring when returned to optimal temperatures [6], the expansion and thriving of snail hosts have attracted considerable attention. Climate warming is considered one of the greatest threats to effective snail control in temperate regions [6–9]. Unlike tropical snails, which are likely to shrink their habitats due to narrow thermal niches, snails in temperate regions are generally considered to expand their habitats with climate warming [10].

The People’s Republic of China (P.R. China) was once one of the countries most affected by schistosomiasis, with over 11 million people infected in the 1950s [11]. *Schistosoma japonicum,* infecting humans and over 40 species of wild and domestic animals [12–15], is the only *Schistosoma* species distributed in China. *Oncomelania hupensis*, as its sole intermediate snail host in China, is a prerequisite for the transmission and spread of schistosomiasis japonica [16]. *Oncomelania hupensis* is an amphibious snail that inhabits and matures on moist soil, and usually has a lifespan of one year [17]. After mating, females lay eggs, and water provides favorable conditions for egg hatching. It has a long reproductive period of 6–7 months, but juveniles commonly occur from April to June, coinciding with warm and rainy conditions [18]. Snail control has been proven to be one of the most effective tools to interrupt the lifecycle of all kinds of *Schistosoma* spp. infecting human beings and WHO encourages member states to integrate snail control with conventional preventive chemotherapy programs to reduce the prevalence and block the transmission of schistosomiasis [19, 20]. In China, snail control has been an essential component of the national schistosomiasis control programmes since the 1950s, alongside treatment of humans and livestock, improvement of sanitation and safe water supply, disease surveillance, environmental modification, etc., leading to substantial progress in reducing both schistosomiasis prevalence and snail habitats [21, 22]. By 2023, the whole country reached the Chinese criteria of schistosomiasis transmission interruption (without new endogenous infection occurring in humans, livestock, and snails in five consecutive years) [23]. Although the area of snail habitats has been dropped from 14,300 km^2^ in the 1950s to 3658 km^2^ by 2024, it is noteworthy that new and re-emerging snail-infested settings have increased, primarily driven by natural disasters such as floods or extreme drought caused by climate change in recent decades [24]. To attain China’s 2030 target for schistosomiasis elimination and sustain the elimination achievements, it is imperative to understand how climate change, especially increased temperatures, affects the distribution of *O. hupensis,* the sole intermediate host of *S. japonicum*. This understanding will thereby provide a scientific basis for formulating climate-adaptive intervention and surveillance strategies against schistosomiasis.

Previous studies have shown that *O. hupensis* is expanding its distribution to occupy suitable ecological niches along several dimensions (i.e., latitude, longitude, and altitude) in response to rising temperatures [25]. However, most studies are based mainly on mean climate, which ignores intraspecific and seasonal variations in climate responses across populations [26, 27]. A further oversight in such studies is that climate warming involves not only a general rise in mean temperature but also shifts in climatic extremes. These stepwise movements are also largely impacted by local habitat availability and microenvironments [28]. It is known that *O. hupensis* expansion is constrained in forests and urban areas [29, 30]. *Oncomelania hupensis* densities exhibit seasonal peaks in different months, depending on the specific habitats that snails colonize [31]. Some research further suggests that climate change and land-use transitions may interact synergistically, leading to a greater effect than would be expected had they acted independently [32]. However, only a few nation-level studies have examined how land use and climate warming interact to influence the population dynamics of *O. hupensis*.

In addition, body size is also a critical trait for understanding the climatic effects on *O. hupensis*. The surface area-to-volume ratio scales with body size, affecting heat retention in ectotherms [33]. For mollusk snails, body size is closely associated with feeding behavior [34], movement [35], and even schistosome infection rate [36]. Body size could thus mediate climatic effects on *O. hupensis* directly by regulating physiological responses [34, 37], or indirectly by modulating inter- and intraspecific competitive interactions [34, 35]. Previous studies have shown variation in *O. hupensis* body size among subspecies in the context of different ecosystems [18], as well as differing body size-thermal tolerance relationships across ectothermic taxa [38], underscoring the importance of investigating this trait. Although there are some experimental studies, field-based evidence and forecasting research on the future change in body size of *O. hupensis* in the context of climate change are scarce.

In this study, we combined 33 years of *O. hupensis* survey data (1990–2022) from the National Parasite Disease Prevention and Treatment Management System and schistosomiasis surveillance site reports to address the following questions spanning individual to ecosystem scales. Here, *O. hupensis* in the same village was considered as a population in line with administrative divisions and the definitions of routine work in China. (1) Individual level: How does body size affect the survival of *O. hupensis* under climate warming? We hypothesized that larger individuals are more likely to survive in unsuitable temperatures. (2) Population level: Is there a spatial correlation between *O. hupensis* density and temperature? And which factors account for spatial variation in population-level temperature responses? We hypothesized that temperature is a key driver of *O. hupensis* density, but the magnitude and direction of this effect vary spatially. Populations that experienced greater historical temperature variability are more likely to survive ongoing climate warming; altitude imposes a geographical constraint on the expansion of *O. hupensis*; and land use adjusts local climate conditions. (3) Ecosystem level: How will *O. hupensis* respond to future climate warming and projected land-use transitions? We hypothesized that waterbodies support higher densities due to favorable habitat conditions, whereas crops support the largest populations because of their projected future expansion and corresponding large-scale irrigation systems.

## Methods

### *Oncomelania hupensis* survey data

We utilized *O. hupensis* survey data mainly collected by the National Parasite Disease Prevention and Treatment Management System. To ensure longer survey time series for as many sites as possible, we further complemented the data from literature [39–41]. These data were collected through annual field surveys conducted mainly from March to April between 1990 and 2022, using multistage sampling. The sampled population spanned latitudes from 20.6 to 35.1˚N, with the northernmost populations near the northern limit. Populations covered longitudes from 97.8 to 122.8˚E and altitudes from − 8.0 m to 4478.0 m. A total of 348,253 records were collected from 34,554 villages across 12 provinces, municipalities and autonomous regions: Anhui (*n* = 2748), Fujian (*n* = 309), Guangxi (*n* = 427), Guangdong (*n* = 269), Hubei (*n* = 5738), Hunan (*n* = 4202), Jiangsu (*n* = 6024), and Jiangxi (*n* = 1553), Shanghai (*n* = 1659), Sichuan (*n* = 4736), Yunnan (*n* = 495), and Zhejiang (*n* = 6394).

*O. hupensis* survey was conducted in combination with systematic sampling and environmental sampling surveys to reduce the probability of missing infestations [42]. Here, we focused on the systematic sampling method, as snail density was estimated based on this approach. According to the *Handbook of Schistosomiasis Control* [43], sampling frames were placed at regular intervals, with each frame covering approximately 0.1 m^2^. The distance between sampling frames ranged from 5 to 50 m, depending on the habitat area or length. All snails within frames were collected, counted, and examined, and the survey dates were also recorded. The density of live snails was calculated using the following formula:

$$Live \,snail\, density \left( {per \,m^{2} } \right) = \frac{Total \,number\, of\, live\, snails}{{Total\, number\, of\, detection \,frames \, \times \,\,0.1}}$$

The annual density growth rate was calculated with the following function:

$$Density \,growth \,rate_{t} = \frac{{Density_{{t_{1} }} - Density_{{t_{2} }} }}{{Density_{{t_{2} }} \left( {t_{1} - t_{2} } \right)}}$$

where ${Density}_{{t}_{1}}$ and ${Density}_{{t}_{2}}$ denote population density at time ${t}_{1}$ and ${t}_{2}$, respectively.

### Land-use data

We used land-use data from China’s Multi-Period Land Use and Land Cover Remote Sensing Monitoring (CNLUCC) database (https://www.resdc.cn/DOI/DOI.aspx?DOIID=54), with a spatial resolution of 1 km^2^. We included five major land-use categories: (1) Crop, (2) Forest, (3) Grass, (4) Waterbody, and (5) Impervious areas. The barren land was excluded because it includes heterogeneous conditions that are difficult to interpret. We obtained future land-use data under three scenarios generated by Zhang et al. [44]. This dataset covers the global domain at 1 km^2^ grid resolution, spanning the period from 2020 to 2100.

For matching data, we created circular buffers with radii of 1 km, 3 km, and 5 km around each site using the buffer function in the R package *raster*. The proportion of each land-use type within each buffer was calculated and extracted, and the land-use type with the highest proportion was defined as the dominant land-use of its corresponding site. We presented results from the 3 km buffer as the primary findings; results from the 1 km and 5 km buffers are presented in the Additional files.

### Climate data

We used the daily minimum, mean, and maximum temperatures as the temperature variable. We downloaded the historical hourly temperature from the ERA5-Land dataset (https://cds.climate.copernicus.eu/datasets/reanalysis-era5-land?tab=download). This data provides land temperature estimates since 1950 at a spatial resolution of 10 km^2^. For each population, we extracted the hourly mean temperature for all years in which *O. hupensis* density data were available. To minimize issues with spatial autocorrelation, we also resampled the temperature data to a 1 km^2^ resolution.

We then calculated the daily temperatures (minimum, mean, and maximum) for each site. We addressed missing data before the calculations. There are 1098 sites (Anhui: *n* = 159; Guangxi: *n* = 4; Hubei: *n* = 120; Hunan: *n* = 69; Jiangsu: *n* = 55; Jiangxi: *n* = 26; Shanghai: *n* = 110; Sichuan: *n* = 390; Yunnan: *n* = 4; Zhejiang: *n* = 161) whose locations did not overlap with the gridded dataset. We matched these sites with the temperatures in their nearest grid cells.

We then calculated the standardized temperature anomaly at each site as the difference between the mean annual temperature for the five years preceding the sample date and the mean annual temperature for 1901–1930 (from the Climate Research Unit Time-Series version 4.09, https://crudata.uea.ac.uk/cru/data/hrg/cru_ts_4.09/), which we used as the baseline. Here, we used the standardized temperature anomaly as a metric to estimate historical climate variability.

$$STA=\frac{{T}_{mean.sample}-{T}_{mean.baseline}}{s{(T}_{mean.baseline)}}$$

where *STA* is the standardized temperature anomaly, ${T}_{mean.sample}$ represents the annual mean temperature over five years preceding the sample date, and ${T}_{mean.baseline}$ represents the annual mean temperatures for the period 1901–1930.

### Laboratory-based controlled temperature experiment

To clarify the association between body size and resilience to non-optimal temperatures, we conducted a controlled-temperature experiment using laboratory-bred adult *O. hupensis*. We first obtained 1000 *O. hupensis* from the Hubei Gongan County laboratory; these snails were maintained at 4 °C until shipment to our laboratory and then acclimated for 5 days under standard conditions (25 °C) before the experiment. The following experiment was conducted according to previous research [45]. Then, snails were randomly assigned to three temperature treatments: 5 ˚C (defined as low Temp), 25 ˚C (defined as control Temp), and 30 ˚C (defined as high Temp), with three replicates per treatment (60 snails per replicate). All tanks were maintained at a suitable relative humidity of 80–95% and a 12:12 h light: dark photoperiod. All snails were continuously maintained for 45 days. To maintain suitable humidity, a sponge was placed beneath the paper on which the snails were kept. Humidity was checked every 3 h, and water was added as needed. Body size and body weight were measured for all living individuals before and after the experiment. Snail survival was recorded every day. Between 8:00 and 9:00 am, snails were gathered to the center of the tanks and evenly sprayed with a small amount of dechlorinated water to maintain target humidity. Food was placed at the edge of each tank, and we added it every day. Between 3:00 and 4:00 pm, motionless snails were then exposed to warm water at 40 °C for 30 s. Snails were defined as dead if no movement was observed after short-term thermal stimulation. And their body sizes were recorded. Snail body size was measured as shell length, defined as the distance from the shell apex to the base [18].

To examine the effect of *O. hupensis* body size on survival, we standardized initial body size using z-scores and categorized it into three classes (Small, Medium, and Large) based on the 33rd and 66th percentiles. Then we grouped snails by treatment and performed log-rank tests to compare the survival differences among the treatments and body-size classes. To analyze the impacts of treatments and body size classes on the survival of *O. hupensis*, we used the R package *survival* to conduct the Cox proportional hazards model, quantifying the impacts of treatments and body size classes on the survival of *O. hupensis* [46, 47]; and tray was also included as a random effect to account for non-independence among snails within the same tray, consistent with previous studies [48].

### Time-window modeling

*O. hupensis* has a well-documented one-year lifespan [18]. Given that high-temperature exposure during *O. hupensis* breeding period may exert a greater impact on its density than annual mean temperature exposure [31, 49], we hypothesized that the climate effects are not uniform throughout the year. To validate this hypothesis, we ran a relative sliding time-window model to provide more detailed information on seasonal variations in its climate responses. For each snail survey, we defined the survey date as the endpoint and traced back 365 days (approximating one full survival cycle of *O. hupensis*). We split this 365-day period into multiple time windows of different lengths. Within each time window, we calculated the minimum, mean, and maximum temperatures, then separately fitted a *density* ~ *temperature* model. Finally, we collected all significant models to identify temperature responses using the following method. For each population, we selected the best-supported model based on the Akaike Information Criterion (AIC) to predict future density.

For more model details, we applied a relative sliding time-window model to each population (snails inhabiting the same village were defined as a single population unit) to detect its temperature response using the R package *climwin* [50]. Specifically, we first defined a time trend (year) model with an intercept as a “baseline model” using the R package *lme4* [51]. Then, we set the parameters of our sliding window approach, including the maximum and minimum window ranges, a step of 3 days, and the aggregate statistics applied to each temperature window (“min”, “mean”, and “max” for minimum, mean, and maximum temperature). Finally, we ran this sliding-window analysis and used a randomization approach to account for multiple-testing-induced false positives. Scaled density was log(x + 1) transformed before the analysis. Each of these model steps is described in more detail below. All models incorporated the year as a covariate to account for long-term temporal trends.

As we tested many time windows, multiple testing introduced inherent risks. To address this issue, we randomized the order of the original data within each population to remove any association between temperature and *O. hupensis* density, and then reran the sliding-window analysis. This randomization procedure was repeated 100 times, and we then compared the observed result with those from the 100 randomizations to estimate the probability of the observed result under the null hypothesis that no relationship exists between temperature and snail density. We used the P_∆AICc_ to assess the probability that an identified time window was a false positive. P_∆AICc_ indicates the probability of obtaining a given ∆AICc in this sliding window analysis when no relationship exists between temperature and snail density.

A relationship between temperature and *O. hupensis* density within a time window was regarded as a valid cue if P_∆AICc_ was ≤ 0.05 (i.e., the chance of such a result occurring in a randomized dataset was ≤ 5%). Time windows ≤ 15 days in duration were also excluded, as such short windows have been shown not to have a biological impact and can produce statistical artifacts [18]. These functions with predicted coefficients would be used to predict future *O. hupensis* densities. Here, we assessed temperature responses using three metrics: sensitivity, duration, and timing. We defined the linear coefficient calculated from a specific population as its sensitivity (a positive coefficient indicates a positive effect, whereas a negative coefficient indicates a negative effect), the time span from window opening to window closing as duration, and the midpoint of the exposure duration as timing.

### General linear mixed-effects modeling

To understand how environmental factors impact temperature responses and population dynamics (i.e., temperature sensitivity, duration, midpoint, density, and density growth rate), we ran a general linear mixed-effects model using the R package *lme4* [51]. In addition to latitude, longitude, and altitude, we included the effects of land use (i.e., crop, forest, grass, waterbody, and impervious areas), historical climate variability (STA), and their interactions as fixed effects. To account for potential spatial autocorrelation in the temperature responses and population dynamics, we used a Matérn correlation function as a random-intercept term, which estimates pairwise correlations between points as a function of their Euclidean distance, using the *spam* package in R [52].

To check the model’s effectiveness, we generated residuals-versus-fitted plots, observed-versus-fitted plots, and Q-Q plots to evaluate whether the residuals followed a normal distribution. The models developed using mean temperature had the highest predictive accuracy, so we conducted the following analysis based on it.

### Projections of the future density of *O. hupensis*

To predict future *O. hupensis* density, we obtained projected daily mean temperatures for 2025–2100 from the National Tibetan Plateau/Third Pole Environment Data Center (https://data.tpdc.ac.cn/zh-hans/data/58cb3cf2-9773–4608-ac84-240228cd3507) under three shared socioeconomic pathways (SSPs): SSP1-2.6, SSP2-4.5, and SSP5-8.5, corresponding to low-, medium-, and high-emission pathways, respectively. Five global climate models (GCMs) of the Coupled Model Intercomparison Project Phase 6 (CMIP6) ensemble were used: CMCC-ESM2, GFDL-ESM4, MPI-ESM1-2-HR, MRI-ESM2-0, and NorESM2-MM. The projected temperature data were estimated based on the ERA5 dataset.

For each GCM, we used general linear mixed-effect models to predict future temperature sensitivities, durations, and midpoints. Using these estimates and the future temperatures, we predicted *O. hupensis* density for each year from 2030 to 2100. Extreme outlier predictions were excluded to ensure reliability. For each population × year combination, we calculated the average predicted density across GCMs to enhance the robustness of predictions. We then summarized snail density for each land-use type using the mean and interquartile range (IQR). We defined *O. hupensis* populations that benefit from climate warming as those whose projected densities exceeded twice the maximum historical density recorded for that population. To further validate the future change in body size, we collected 240 wild *O. hupensis* from a region predicted to harbor populations benefiting from climate warming and measured their body sizes in 2023.

All analyses were performed using R version 4.4.2 (R Foundation for Statistical Computing, Vienna, Austria).

## Result

### Individual traits of *O. hupensis* under different temperature exposure

**Fig. 1:**
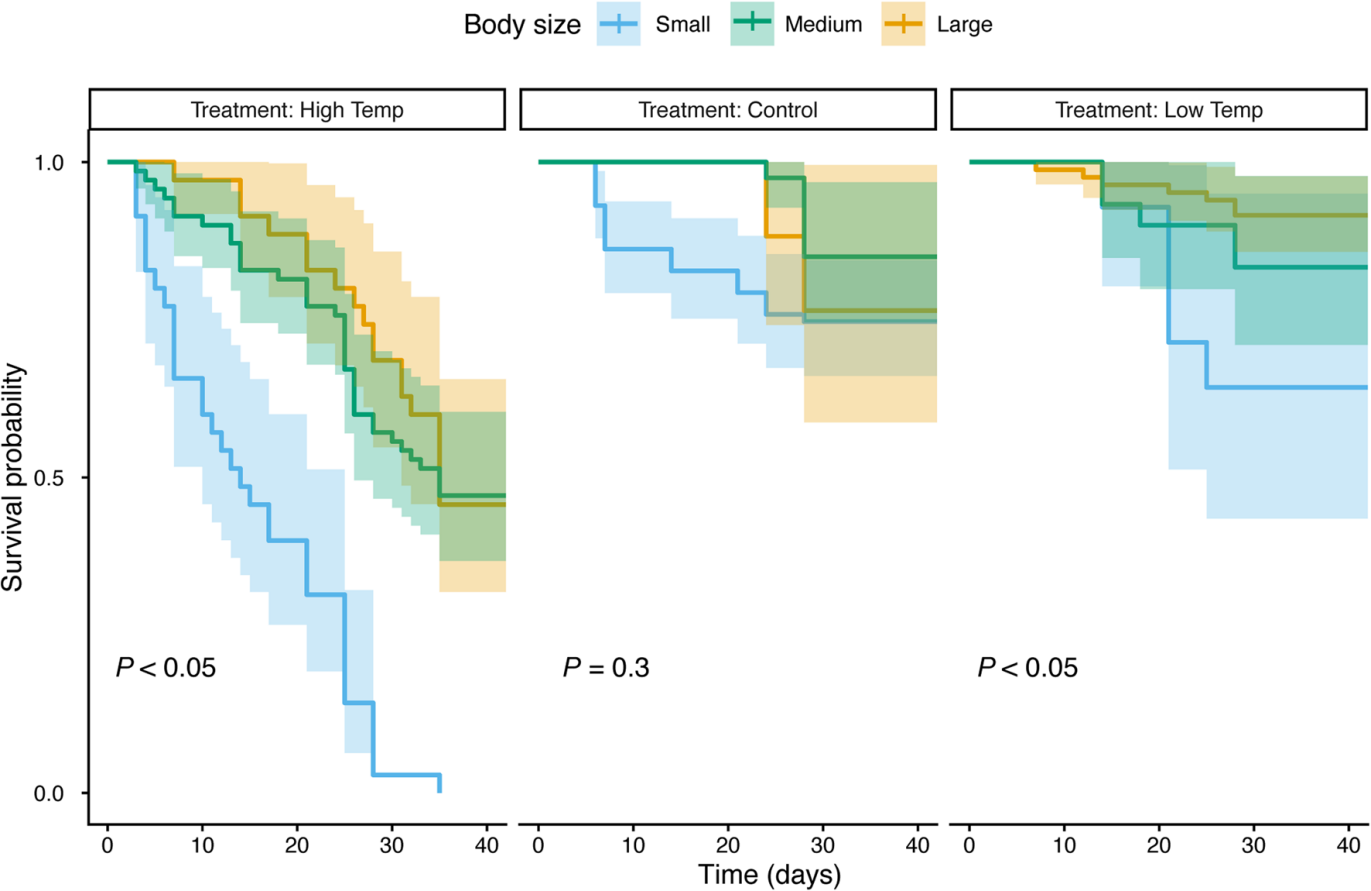
Effects of temperature treatments on the survival of adult *Oncomelania hupensis*. Survival curves of adult *Oncomelania hupensis* with small, medium, and large body sizes in groups treated by Low Temp, Control, and High Temp over the 45-day experimental period. Survival differed significantly among treatments (Log-rank test, *P* < 0.05)

In the Control group, mortality was low, with 180 snails at day 0 decreasing slightly to 163 at day 45. In contrast, the High Temp group exhibited pronounced mortality, declining from 180 to 89 snails over the same period, while the Low Temp group showed intermediate mortality, with 148 individuals surviving at day 45 (Additional file 1). Cox proportional hazards models showed there are significant effects of temperature, body size, and treatment × size on snails survival. Snails in the High Temp group showed a significantly higher hazard of mortality than the control group (*χ*^*2*^ = 51.1, *P* < 0.05; Additional file 2). Body size was also significantly associated with snail survival (*χ*^*2*^ = 3.2, *P* < 0.05; Additional file 2), and a marginal interaction between high temperature and body size was detected (*χ*^*2*^ = 3.1, *P* < 0.1; Additional file 2). In the High Temp group, small snails exhibited the lowest survival, reaching 2.9% (95% *CI* 0.4–19.7%), followed by moderate (47.1%, 95% *CI* 36.8–60.4%) and large snails (45.7%, 95% *CI* 31.9–65.6%). In the Low Temp group, small snails also showed the lowest survival (64.3%, 95% *CI* 43.5–95.0%), whereas large snails had the highest survival (91.6%, 95% *CI* 85.8–97.7%), followed by moderate snails (83.3%, 95% *CI* 71.0–97.8%). In the Control group, moderate snails exhibited the highest survival (85.0%, 95% *CI* 74.6–96.8%), compared with small (74.7%, 95% *CI* 66.1–84.4%) and large snails (76.5%, 95% *CI* 58.7–99.5%) (Fig. 1, body weight changes presented in Additional file 3).

### Seasonal temperature responses in *O. hupensis*

**Fig. 2:**
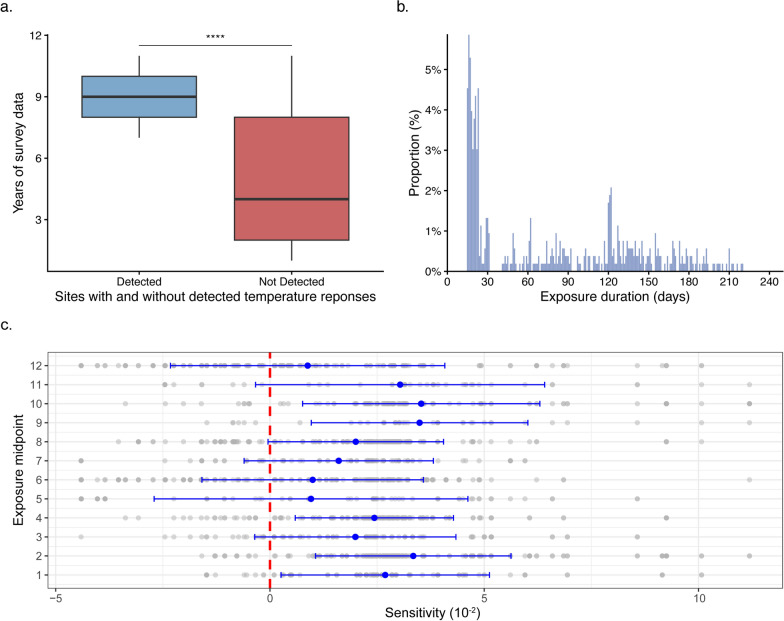
Characteristics of temperature responses of *Oncomelania hupensis* detected in China. **a** Relationship between survey length and detection of temperature responses. **b** Proportions of temperature sensitivity directions. **c** Distribution of temperature exposure duration; **d** Relationship between the midpoint and sensitivity to mean temperature

Among the data collected from 34,554 villages across 12 provinces, only populations with more than 10 years of available survey data (*n* = 1123) were included to ensure model validity. A significant temperature response was identified in 74.2% of the filtered populations (833/1123), with the geographic distribution of these sites shown in Additional file 1. Temperature responses were detected more frequently in populations with longer records (*t* = 19.7, *P* < 0.001; Fig. 2a), suggesting that the failure to detect a temperature response after randomization is due to insufficient data rather than the absence of such a response. Of the 833 populations with a temperature response, 92.0% showed positive temperature sensitivity, with corresponding durations ranging from 23 to 220 days. (Fig. 2b).

Nearly half of the population exhibited significant temperature sensitivities during the coldest season (January–February: 46.7%), with a value of 2.7 × 10^–2^ (95% *CI* 3.0 × 10^–3^–5.1 × 10^–2^) and 3.3 × 10^–2^ (95% *CI* 1.1 × 10^–2^–5.6 × 10^–2^), respectively. Significant temperature sensitivity was also detected during the spring and autumn breeding periods (April: 28.0%; September–October: 23.2%), with effects of 2.4 × 10^–2^ (95% *CI* 6.0 × 10^–3^–4.3 × 10^–2^) and 3.5 × 10^–2^ (95% *CI* 2.0 × 10^–3^–6.8 × 10^–2^), respectively. (Fig. 2c) Seasonal variations in temperature sensitivity were additionally observed for both minimum and maximum temperatures. (Additional file 4).

### Environmental factors drive the variation of temperature responses

**Fig. 3:**
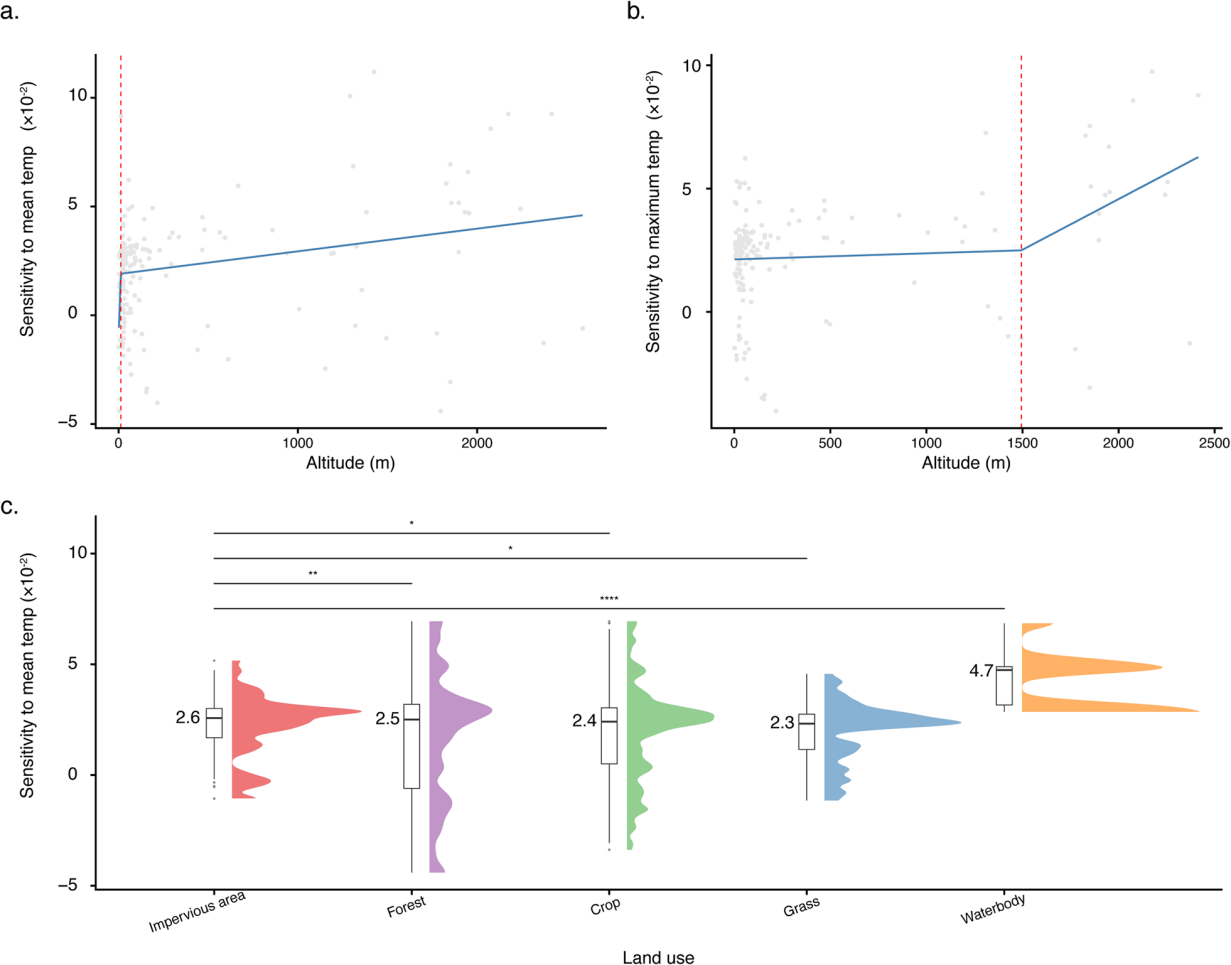
Geographical constraints for temperature sensitivity in *Oncomelania hupensis*. **a** Segmented altitudinal patterns of sensitivity to mean temperature. **b** Segmented altitudinal patterns of sensitivity to maximum temperature. **c** Differences in mean temperature sensitivity among land-use types

A distinct pattern along the latitudinal and altitudinal gradients was observed. Populations at higher latitudes and altitudes presented stronger maximum temperature sensitivity (latitude: *β* = 2.1 × 10^–3^, 95% *CI* 1.7 × 10^–3^–2.4 × 10^–3^; altitude:* β* = 1.6 × 10^–5^, 95% *CI* 1.4 × 10^–5^–1.7 × 10^–5^; Additional file 5). Population density increased with increasing latitudes but decreased at higher altitudes (latitude:* β* = 1.4 × 10^–2^, 95% *CI* 8.0 × 10^–3^–2.0 × 10^–2^; altitude: *β* = − 2.1 × 10^–4^, 95% *CI* − 2.4 × 10^–4^to − 1.9 × 10^–4^; Additional file 6). We further applied segmented linear models to identify altitudinal breakpoints in temperature sensitivity. Mean temperature sensitivity rose rapidly with increasing altitude below 13.7 m (95% *CI* 7.0–14.0), whereas the rate of increase slowed at higher altitudes. (Fig. 3a) In contrast, maximum temperature sensitivity increased slightly with altitude below 1663.0 m (95% *CI* 1493.9–1775.0), whereas it increased rapidly above this threshold. (Fig. 3b; Additional file 7).

For different land-use types, *O. hupensis* in waterbody-dominated areas had the highest density growth rate (*ß* = 1.6 × 10^–1^, 95% *CI* 1.1 × 10^–1^–2.2 × 10^–1^), whereas those in other land uses had nearly the same level. (Fig. 3c; Additional file 6) In crop‐dominated areas, the responses of *O. hupensis* to minimum and maximum temperatures primarily occurred in June, whereas responses to mean temperatures were concentrated later, peaking in August. *O. hupensis* inhabiting waterbodies showed that their minimum and mean temperature responses mainly occurred in April, with the maximum temperatures observed in June (Additional file 8). Notably, *O. hupensis* populations in crops were the most sensitive to land-cover change: a 1% increase in crop cover was associated with a 0.2 increase in population density. This result remained robust when the analysis was conducted using 1 km and 5 km buffers (Additional file 9).

Additional model results indicated that variations in temperature responses also depended on the magnitude of historical climate variability. Populations with larger standardized temperature anomalies showed weaker sensitivity to minimum and mean temperatures (minimum:* ß* = − 1.0 × 10^–2^, 95% *CI* − 1.6 × 10^–2^ to − 3 × 10^–3^; Additional file 10; mean: *ß* = − 2.1 × 10^–2^, 95% *CI* − 2.7 × 10^–2^to − 1.4 × 10^–2^; Additional file 6), but stronger sensitivity to maximum temperature (maximum: *ß* = 1.2 × 10^–1^, 95% *CI* 6.6 × 10^–2^–1.8 × 10^–1^; Additional file 5). *O. hupensis* inhabiting areas with more intensive climate variability were observed to have higher densities and growth rates (density: *ß* = 0.3, 95% *CI* 0.2–0.4; growth rates: *ß* = 0.3, 95% *CI* 0.1–0.5; Additional file 6). Furthermore, synergistic interactions between land use and historical climate variability were associated with differences in temperature responses across land-use types. A warming scenario equivalent to one standard deviation of baseline temperature variation (STA = 1.0) resulted in a 52.1% and 9.7% increase in *O. hupensis* densities in waterbodies and forests, respectively, compared to those in impervious areas. At the same level of climate warming, *O. hupensis* densities in grass and crops decreased by 56.4% and 15.3%, respectively. (Additional file 11) General linear mixed-effects models developed using mean temperature had a higher predictive accuracy. (Additional file 12).

### Prediction for future *O. hupensis* density with changes in climate and land-use

The fraction of the population benefiting from climate warming is projected to keep decreasing in the future. In 2100, these proportions will decline to 24.6% (205/833) under SSP1-2.6, 21.5% (179/833) under SSP2-4.5, and 19.0% (158/833) under SSP5-8.5, respectively (Additional file 13). Most of these populations are projected to be distributed in the regions around Poyang Lake, whereas only a small proportion is expected to be distributed in the upper reaches (Sichuan province and Yunnan province). The body size of adult *O. hupensis* collected from 8 sites in these regions ranged from 6.4 to 9.2 mm, with significant variation observed across sampling sites. Among the sampled provinces, snails from Hubei Province showed the largest mean body size (8.8 mm), followed by Anhui Province (8.2 mm) and Jiangxi Province (7.7 mm). By contrast, snails from Yunnan Province and Sichuan Province showed the smallest mean body sizes, at 6.6 mm and 6.9 mm, respectively.

**Fig. 4:**
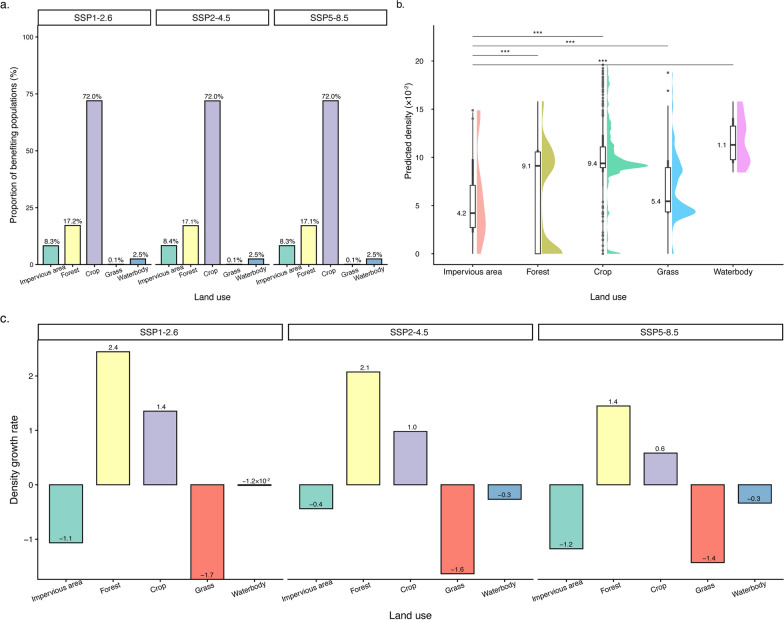
Land-use-specific population responses of *Oncomelania hupensis* to climatic warming under different SSPs. **a** Proportion of populations benefiting from climatic warming across land-use types under SSP1-2.6, SSP2-4.5, and SSP5-8.5. **b** Comparison of predicted population density among land uses under SSP1-2.6. **c** Predicted density growth rate across land-uses under SSP1-2.6, SSP2-4.5, and SSP5-8.5

Future land-use transition projections indicate that croplands would experience the largest increase in land cover, accounting for 72.0% of the population benefiting from climate warming. These differences remained robust across SSPs and GCMs. (Fig. 4a, Additional file 14) Predicted density differs significantly among land-use types (*χ*^*2*^ = 73.5, *P* < 0.05). Under SSP1-2.6, waterbodies are projected to support the highest population densities (1.1 × 10^–1^, IQR: 9.3 × 10^–2^–2.6 × 10^–1^), while impervious dominant areas are predicted to have the lowest densities (4.2 × 10^–2^, IQR: 0–6.1 × 10^–2^, Fig. 4b). This ranking was consistent across all SSPs and GCMs. (Additional file 15) Across all SSPs and GCMs, grass and impervious-dominated areas consistently showed the lowest predicted density growth rates. (Fig. 4c, Additional file 16).

## Discussion

We investigated the temperature response of *O. hupensis* using long-term survey data (1990–2022). To our knowledge, this is the first study to examine the impacts of climate warming on *O. hupensis* using daily temperature variation. As expected, temperatures during the coldest season and breeding seasons significantly affected the density of *O. hupensis*. This result underscored the importance of seasonal specialists in understanding population-level temperature sensitivities to climate warming. Consistent with previous studies, temperature sensitivity was associated with historical temperature variability, which may filter out snails with narrow thermal tolerances [53]. Geographical barriers and land use also either limit or facilitate the population density of *O. hupensis* under climate warming [54–56]. We predicted that crops would harbor the majority of *O. hupensis* populations due to ongoing agricultural expansion, which is accompanied by irrigation system development [57]. Model projections showed that most *O. hupensis* populations were concentrated around Poyang Lake, the largest freshwater lake in China. We further observed that the mean body size of wild snails from this region was 8.2 mm [34].

A large proportion of the populations involved in the model showed positive temperature responses, indicating that temperature is a key driver of *O. hupensis* expansion. Nevertheless, our findings also highlight the vulnerability of numerous populations to climate warming. At the individual level, model projections suggested that snail populations around Poyang Lake are more likely to persist in the future. Field snail sample observation further indicated that wild snails sampled from these regions are more likely to grow large than those sampled from Sichuan province and Yunnan province, due to altitudinal gradients [58]. We also observed that snails from sites predicted to benefit from climate warming exhibited larger body sizes than the regional mean. In Hubei, Anhui, and Jiangxi, mean body sizes were 8.8 mm, 8.2 mm, and 7.7 mm, respectively, all above the regional mean (7.5 mm). The snails from Yunnan and Sichuan were smaller (6.6 mm and 6.9 mm), but still exceeding their regional mean (6.4 mm). In addition, we found that snails with larger body sizes showed higher survival under both low- and high-temperature conditions due to greater thermal tolerance, consistent with previous findings in ectotherms [59, 60]. However, according to the temperature-size rule, snails tend to grow smaller under high temperature [34], and we emphasized the vulnerability of *O. hupensis* populations under climate warming. At the population level, negative latitudinal and altitudinal trends in minimum-temperature responses were observed, and the majority of temperature responses were detected during the coldest months of the year. These results suggested that minimum temperature may serves as a threshold constraint rather than a direct driver of population growth [61, 62], but more research is needed to validate it. At the ecological level, we found that the number of populations benefiting from climate warming declines with increasing greenhouse gas emissions scenarios. This suggests that the positive effect of climate warming on *O. hupensis* is not indefinite; once temperature exceeds a certain threshold, snail density will decrease. We infer that suitable habitats for *O. hupensis* will shrink in low-latitude regions while likely expanding towards higher-latitude or cooler areas under future climate warming. Due to the buffering effect of waterbodies, regions around Poyang Lake were identified as risk hotspots across all scenarios, consistent with previous studies [63]. This highlights the need for enhanced surveillance and targeted control efforts in these regions. In addition, geographical barriers appear to constrain the expansion of *O. hupensis*, populations at higher altitudes exhibited lower densities despite showing greater temperature sensitivity.

Historical climate variability is an additional important driver of heterogeneity in temperature responses [64]. Populations experiencing greater climate variability exhibited weaker sensitivity to minimum and mean temperatures yet heightened sensitivity to maximum temperatures, may suggesting that extreme temperatures may exert a more pronounced driving effect on population growth than mean temperatures, as previous study concluded[62], but further studies are needed. Synergistic effects between land-use and climate variability further indicate that land-use provides a microclimatic buffer that modulates population temperature responses [53, 65]. *O. hupensis* control measures include a series of environmental modification projects, including land management, amelioration of waterlogged fields and ecological land conservation, etc. [66]. A warming scenario equivalent to one standard deviation of baseline temperature variation (STA = 1.0) resulted in a 9.684% increase in *O. hupensis* density in forests, but the predicted density in forests remained relatively low, similar to that in urban areas. *O. hupensis* control and schistosomiasis prevention forest, urban areas have been shown to have a negative impact on the snails. These land use transitions that negatively affect *O. hupensis* usually be used in schistosomiasis prevention and *O. hupensis* control. China launched forestry schistosomiasis control project in 2006, and planted a total of 5.2 billion m^2^ of *O. hupensis* control and schistosomiasis prevention forest in 10 years [67]. On one hand, the programs were designed to deteriorate *O. hupensis* breeding environment by creating forests [68]. Previous studies have shown that enzyme levels in *O. hupensis* in the environments of the forestry schistosomiasis control project were different from those in the snail natural habitats, indicating that snail enzyme and energy metabolism might be interfered with the forests [69]. On the other hand, bioactive compounds produced by plants can exert allelopathic effects on *O. hupensis*, thereby achieving biological snail-inhibition [70]. Since the start of this program, the density of snails had decreased by 89.9%, and the density of infected snails had decreased by 95.8% in 10 years [67]. In addition, the project also increased the forest coverage, enhanced water conservation and reduced soil erosion as rainwater could be intercepted by tree canopy and soils could be fixed by tree roots [71]. In recent years, there has been research that found that the negative effect of urban areas is declining [29]. Our results suggested the similar pattern may also happen in forest, but further research is needed. At the same level of temperature rise, snail populations in waterbodies exhibited increased density. The crops were predicted to undergo the most extensive expansion in China [44], which is accompanied by the development of irrigation systems. A close correlation between the distribution of *O. hupensis* and water canal systems has been observed in endemic regions. Frequent human water contact along canals and ditches further increases the risk of schistosomiasis transmission, resulting in higher numbers of patients [72]. The integration of water resource development and management with effective snail control measures could yield twice the result with half the effort [73, 74]. Therefore, schistosomiasis control considerations should be included in discussions at the early stages of irrigation scheme design [75].Snail habitats are often found in small ditches surrounding cropland in both plains and terraced areas [76]. The conversion of rice paddies to dryland in some mountainous areas showed good effectiveness in snail control and schistosomiasis transmission interruption [77].

As with any correlational study, we cannot infer causal effects; moreover, this study uses a spatial rather than a temporal analysis, and a key limitation of spatial analysis is that it cannot account for time lags in the effects of environmental change on growth rates. Furthermore, the majority of our data originated from the post-2000 period; therefore, our analysis may not comprehensively capture the historical dynamics of *O. hupensis* populations. In addition, we did not include water temperature, as long-term daily water temperature data were not available. Instead, we included waterbody land-use to partially account for its potential effects. In the experiment, we used short-term thermal stimulation rather than the pin-puncture method used in previous studies to assess snail survival, in consideration of animal welfare. Due to the snails used in the experiment being transported at 4 °C, we first acclimated them at 25 °C before the normal experiment. In this study, populations were defined at the village level, which may not fully capture smaller-scale population differentiation. Several analyses were conducted to enhance the robustness of our results. These included: (1) repeating the analysis across five GCMs to account for climate model uncertainty; (2) testing different SSPs to evaluate the sensitivity of predictions to socio-economic pathways; (3) performing outlier filtering to ensure that extreme values did not drive observed patterns.

Despite the limitations of correlational analyses, our results showed a clear and consistent association between *O. hupensis* temperature response and the interaction of land use and climate warming. Future work incorporating phenotypic traits and genomic analyses will provide more detailed insights into the impacts of climate change on *O. hupensis*. We did not include other aspects of climate, such as precipitation, here. In fact, temperature is considered a key climatic variable influencing the development of *O. hupensis* populations. Nevertheless, studies that incorporate additional climatic variables and finer-scale land-use and/or land-use intensity data may yield further insights. In addition, although our analysis focus on *O. hupensis* in China, the modeling framework developed in this study can also be applied to other climate-sensitive host–parasite systems, providing an early surveillance strategy to inform targeted mitigation efforts under climate change.

## Conclusions

The findings in our research, consistent with previous studies [17], show that the increase in *O. hupensis* density is most pronounced in northern China, where recent climate warming represents a larger departure from historical seasonal and interannual temperature variability. Agriculturalization intensifies the impacts of *O. hupensis* expansion, whereas grass- and impervious-dominated areas act as barriers. Therefore, a more precise schistosomiasis surveillance system is needed for the future. From a One Health perspective, our results underscore the interconnectedness of environmental change, snail ecology, and human health. The expansion and density increase of *O. hupensis* driven by climate warming and land-use change not only influence snail populations but also elevate the risk of schistosomiasis transmission to humans and livestock. Integrating ecological monitoring, climate modeling, and public health strategies is essential to anticipate potential outbreaks and implement timely interventions. This holistic approach highlights that effective disease control requires considering human, animal, and environmental health simultaneously. Understanding the trend of *O. hupensis* expansion and population dynamics will benefit both endemic and non-endemic areas. In all scenarios, more than 20% of the population increased its density. To eliminate schistosomiasis and consolidate the achievements obtained in China, surveillance and mitigation of the impacts of climate warming and land use on schistosomiasis, particularly intermediate snail hosts, should be strengthened to provide insights for precise intervention and resource allocation.

## Supplementary Information

Supplementary Material 1. The survival analysis of the controlled temperature experiment.

Supplementary Material 2. Results from the Cox regression with and without temperature × body size interactions.

Supplementary Material 3. Comparison of body weight before and after the experiment.

Supplementary Material 4. Relationship between the midpoint and sensitivity (results from minimum and maximum temperature).

Supplementary Material 5. Result from general mixed function (maximum temperature).

Supplementary Material 6. Result from general mixed function (mean temperature).

Supplementary Material 7. Results from Segmented Linear Models.

Supplementary Material 8. Distribution of exposure midpoint.

Supplementary Material 9. Correlation between land use cover and *Oncomelania hupensis* density.

Supplementary Material 10. Result from general mixed function (minimum temperature)

Supplementary Material 11. Result from the general mixed model with interaction between land use and standardized temperature anomaly.

Supplementary Material 12. Model diagnosis results of general linear mixed-effect models.

Supplementary Material 13. The population benefits from global warming under SSP1-2.6, SSP2-4.5, and SSP5-8.5.

Supplementary Material 14. Distribution of population benefiting from climate warming across the land-uses under SSP1-2.6, SSP2-4.5, and SSP5-8.5.

Supplementary Material 15. Predicted density across land uses under SSP1-2.6, SSP2-4.5, SSP5-8.5.

Supplementary Material 16. Predicted growth rate across land uses in GCMs.

## Data Availability

Data and codes are available at https://www.scidb.cn/en/preview?dataSetId=6ad71671fa3f4fb0adc876aed01abbca&version=V1.

## Abbreviations

AIC: Akaike information criterion
CI: Confidence interval
CMIP6: Coupled Model Intercomparison Project Phase 6
CNLUCC: China’s Multi-Period Land Use and Land Cover Remote Sensing Monitoring
GCMs: Global climate models
IQR: Interquartile range
P.R. China: People’s Republic of China
SSPs: Shared socioeconomic pathways
STA: Standardized temperature anomaly
WHO: World Health Organization
