# Supplementary material for: Impact of climate warming on Oncomelania hupensis in China: multi-scale evidence
Source: Infect Dis Poverty. 2026 Jul 3;15:76. doi: 10.1186/s40249-026-01475-0 (PMC13330383; doi:10.1186/s40249-026-01475-0)
Supplement: Supplementary file 1 — Supplementary Material 1. The survival analysis of the controlled temperature experiment. [file 40249_2026_1475_MOESM1_ESM.docx]

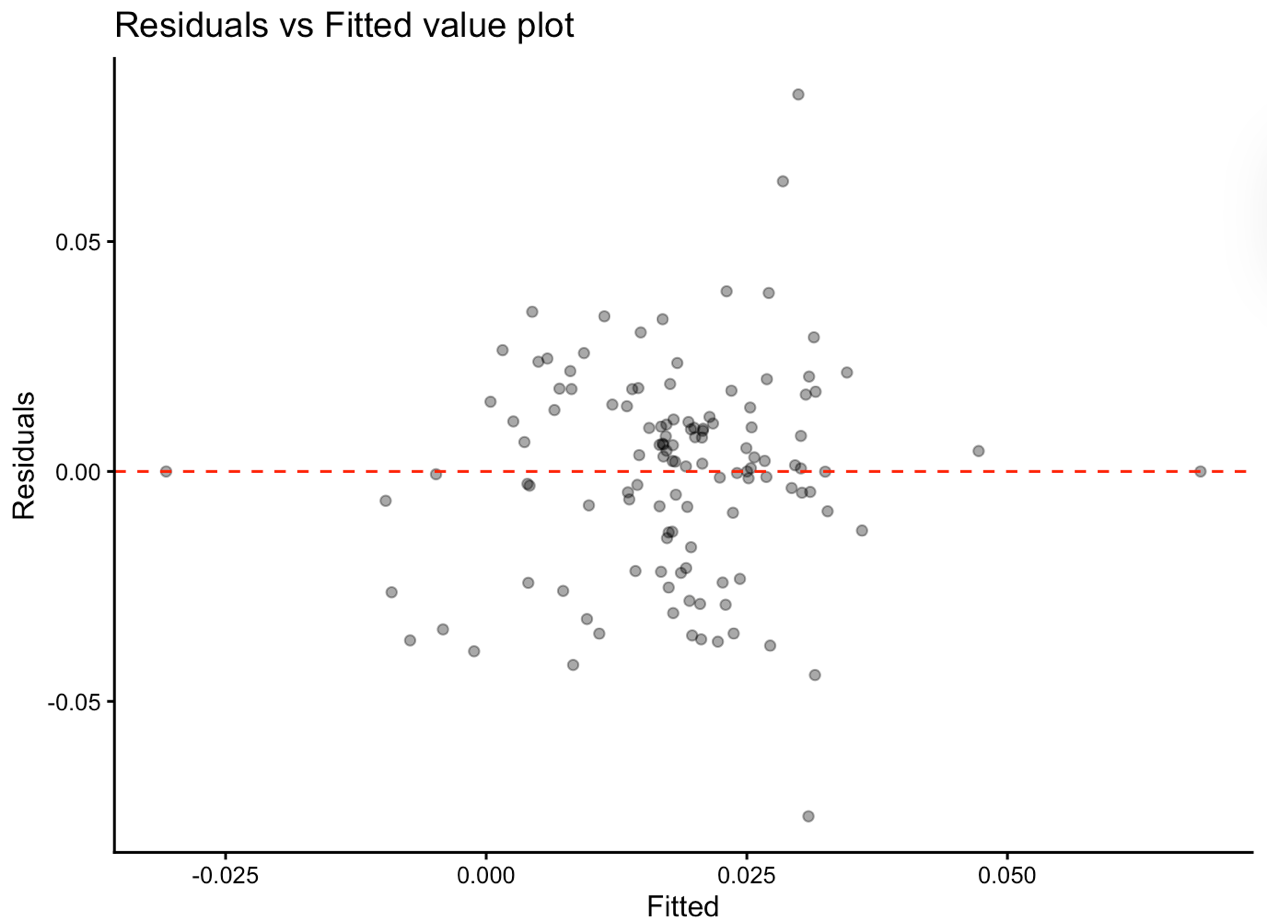


**Figure A1: Model diagnosis result from the Residuals vs fitted value plot (Sensitivity)**


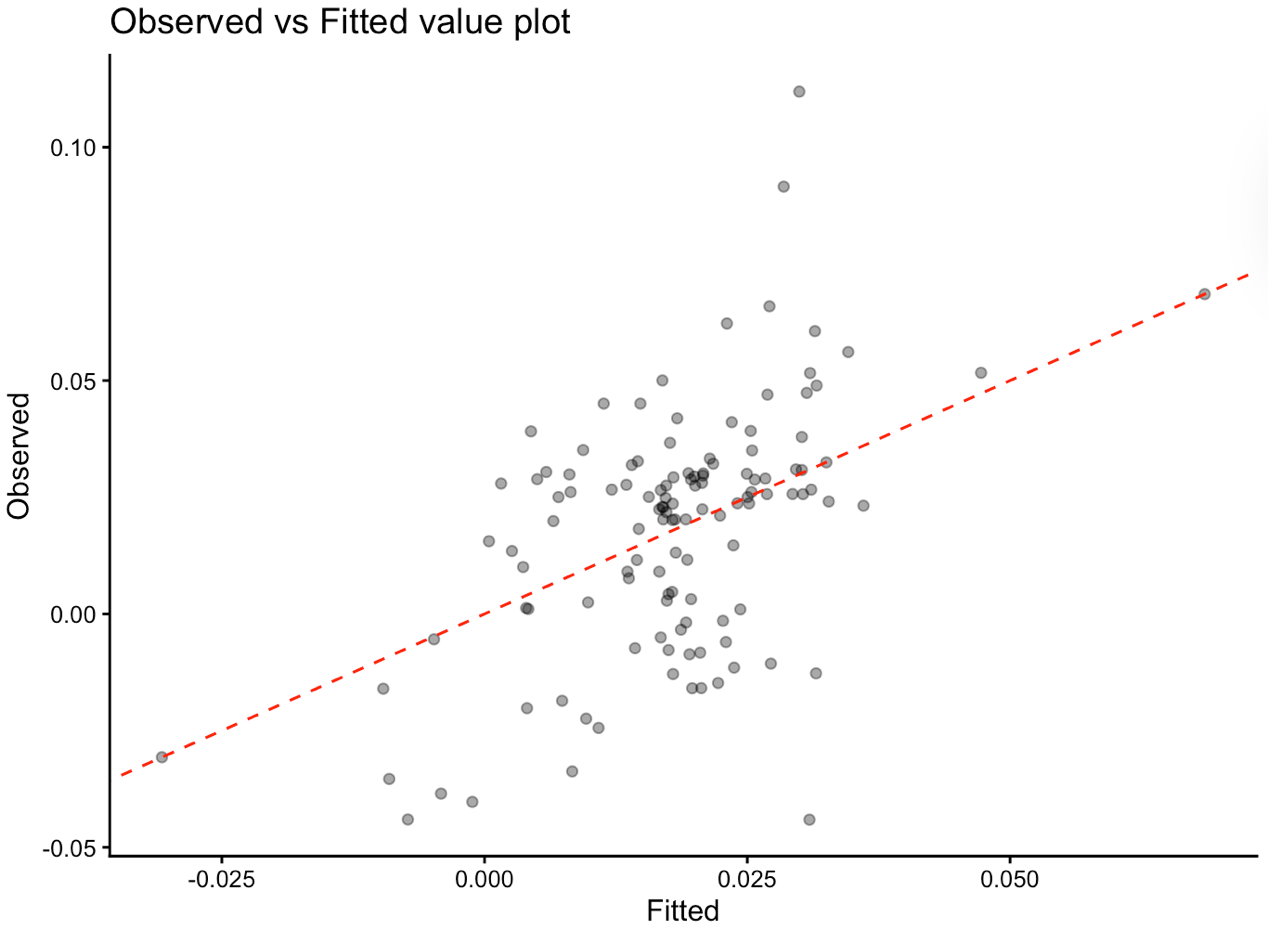


**Figure A2: Model diagnosis result from the Observed vs fitted value plot (Sensitivity)**


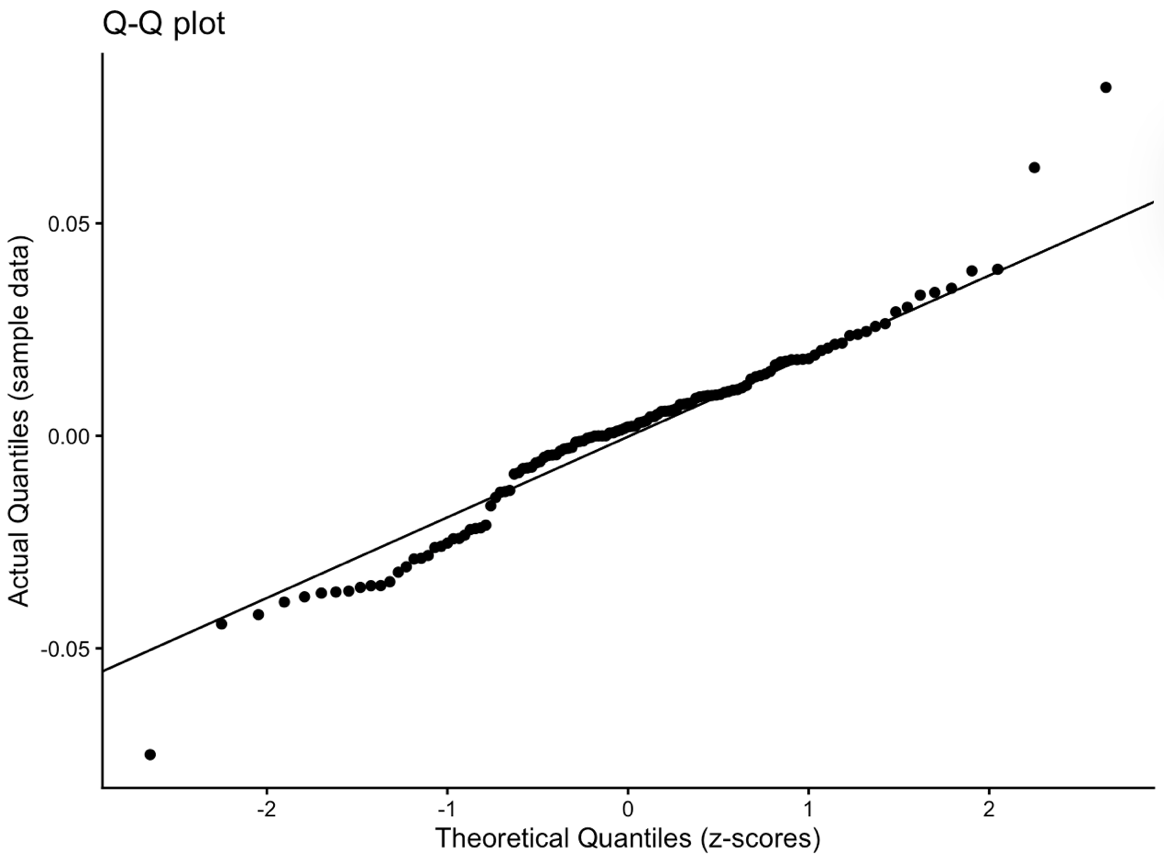


**Figure A3: Model diagnosis result from Q-Q plot (Sensitivity)**


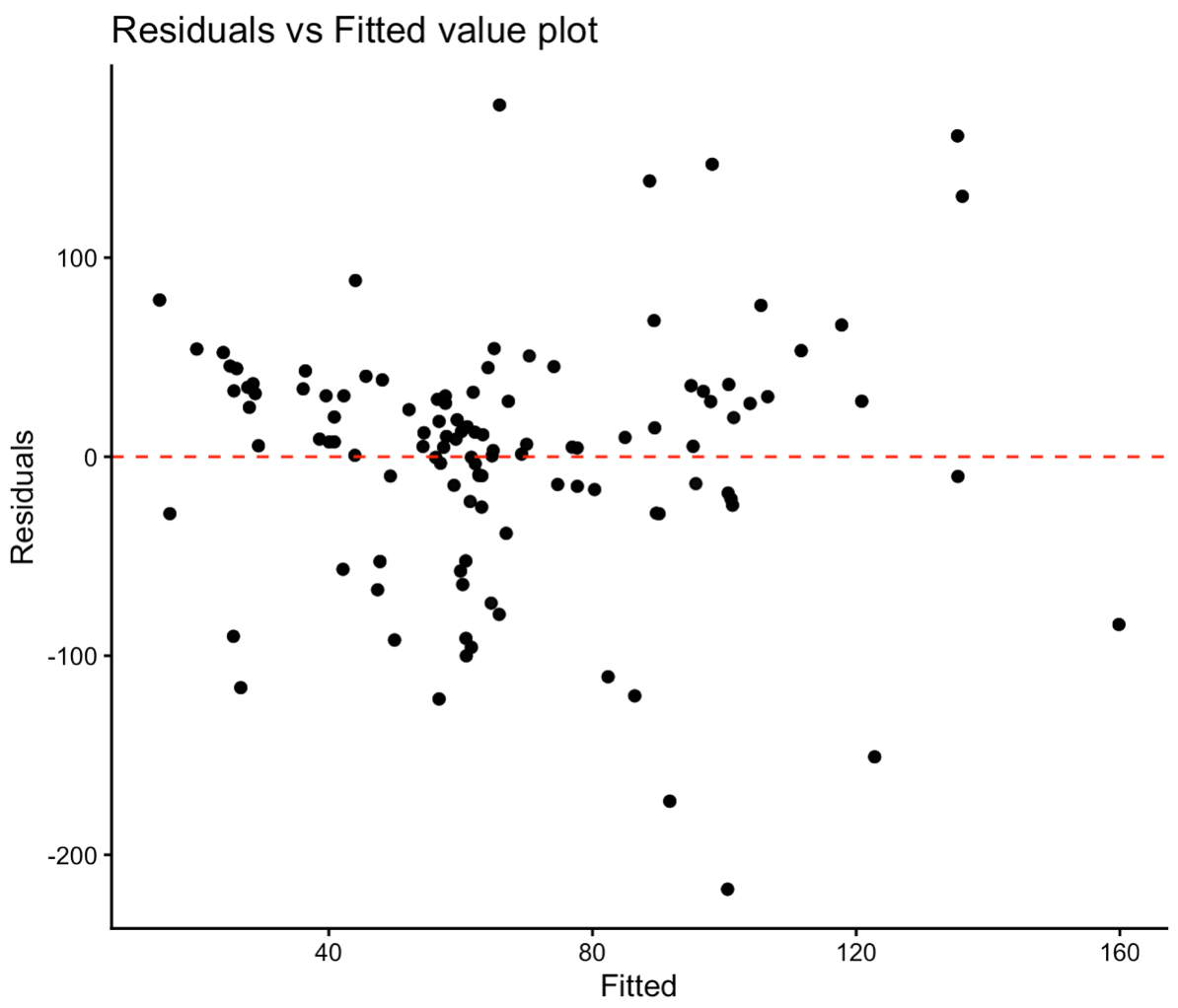


**Figure A4: Model diagnosis result from the Residuals vs fitted value plot (duration)**


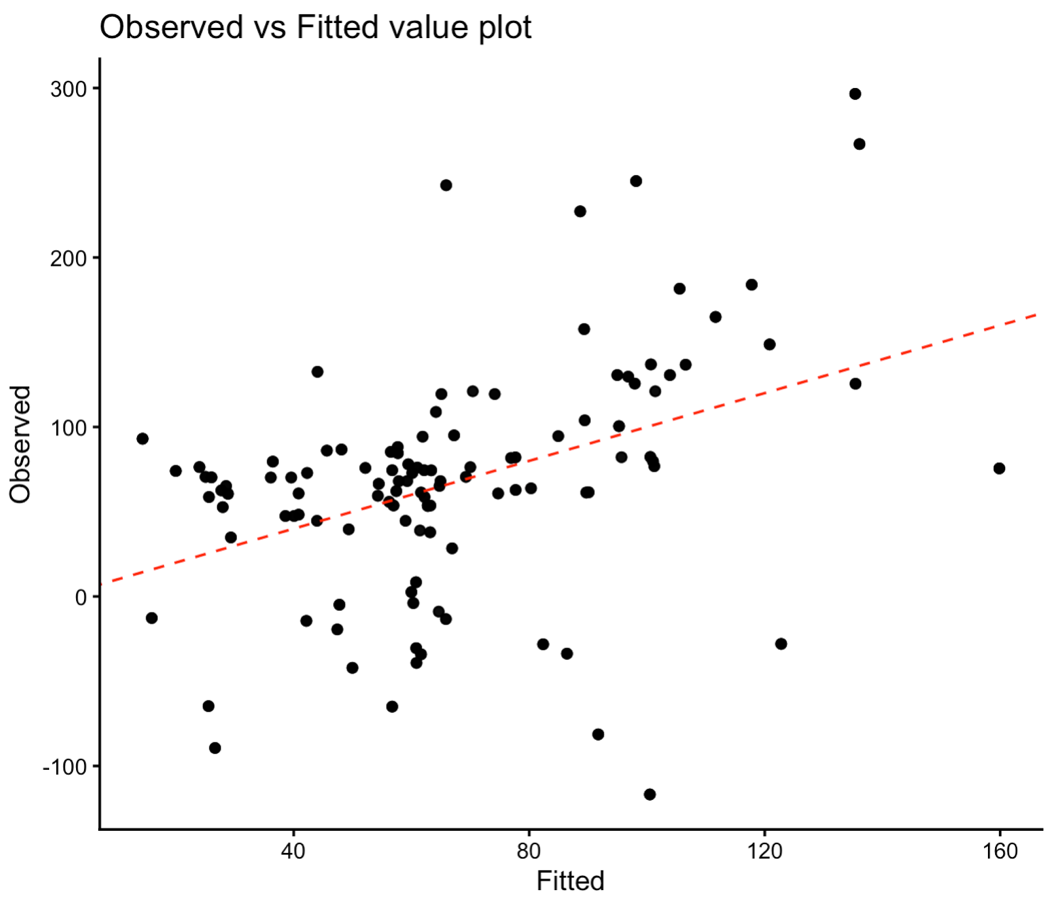


**Figure A5: Model diagnosis result from the Observed vs fitted value plot (duration)**


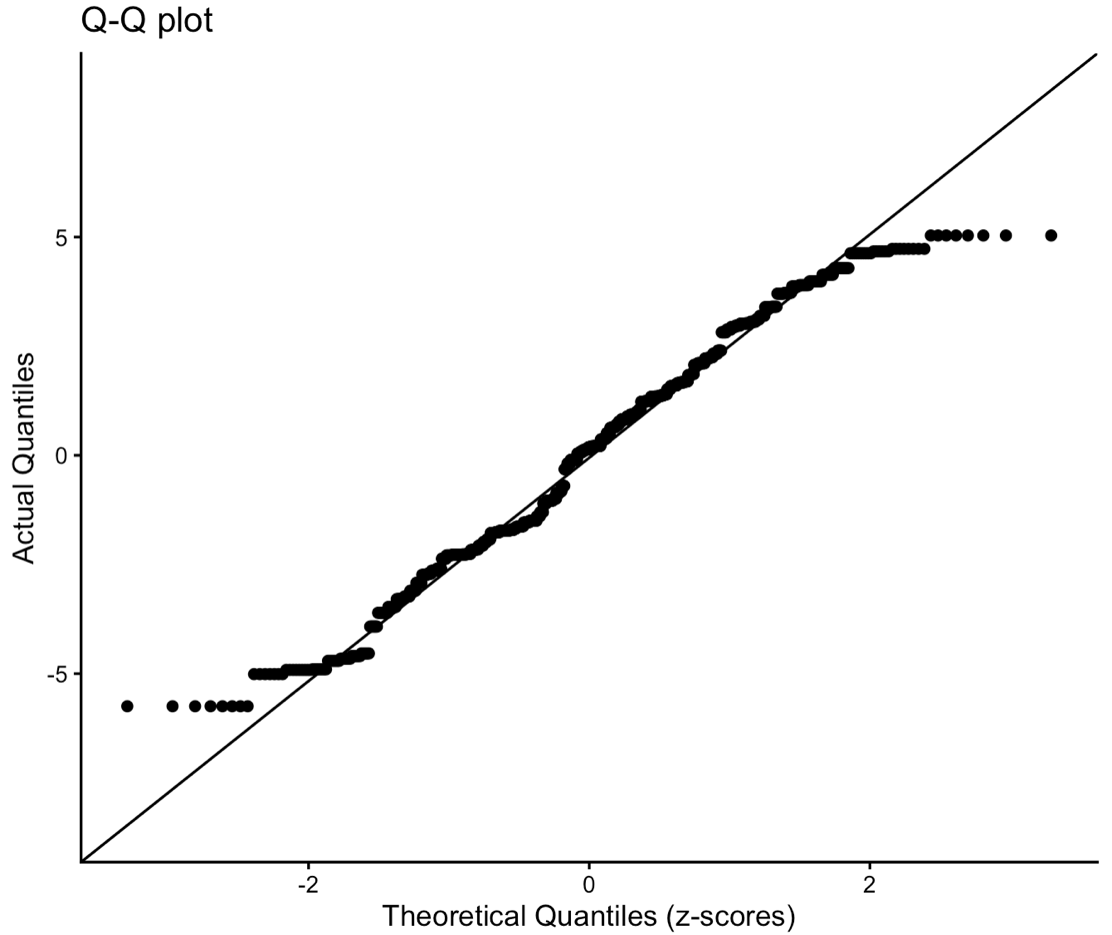


**Figure A6. Model diagnosis result from Q-Q plot (duration)**


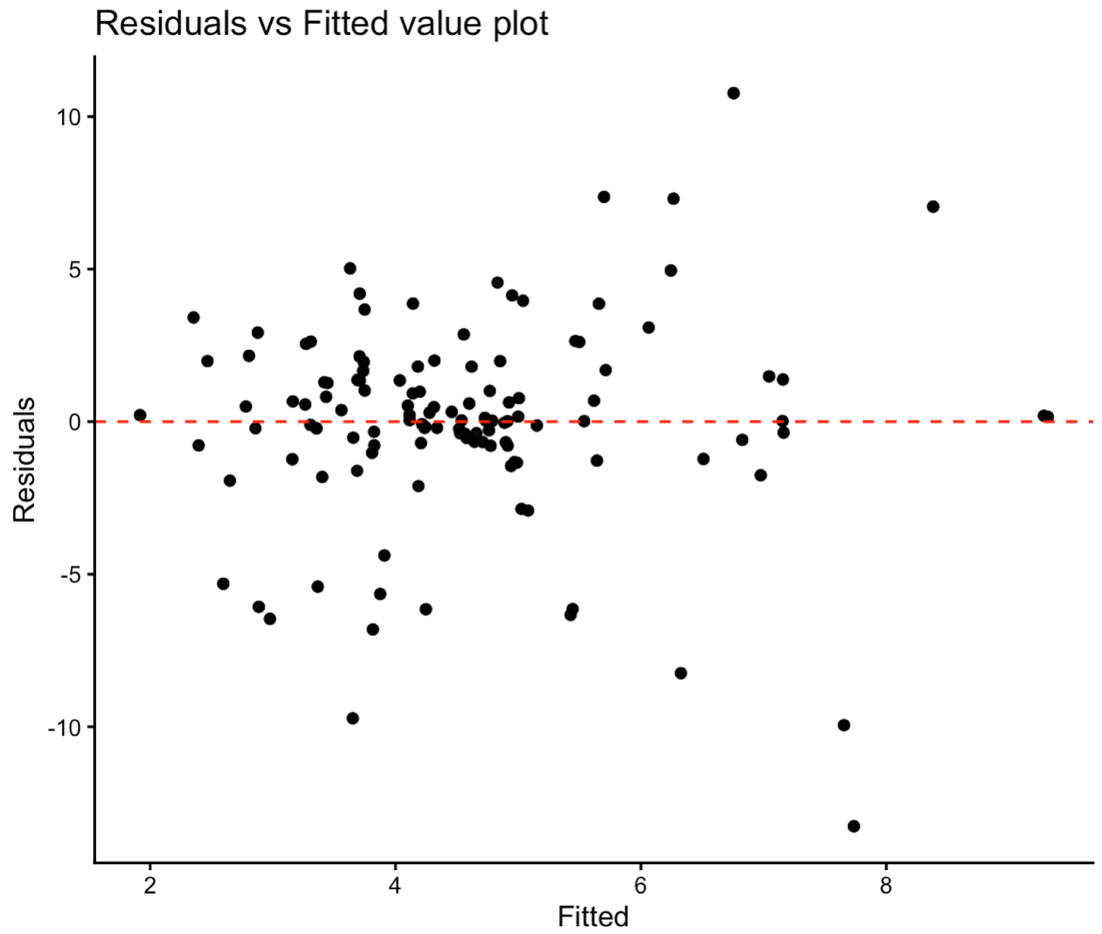


**Figure A7. Model diagnosis result from the Residuals vs Fitted Value plot (midpoint)**


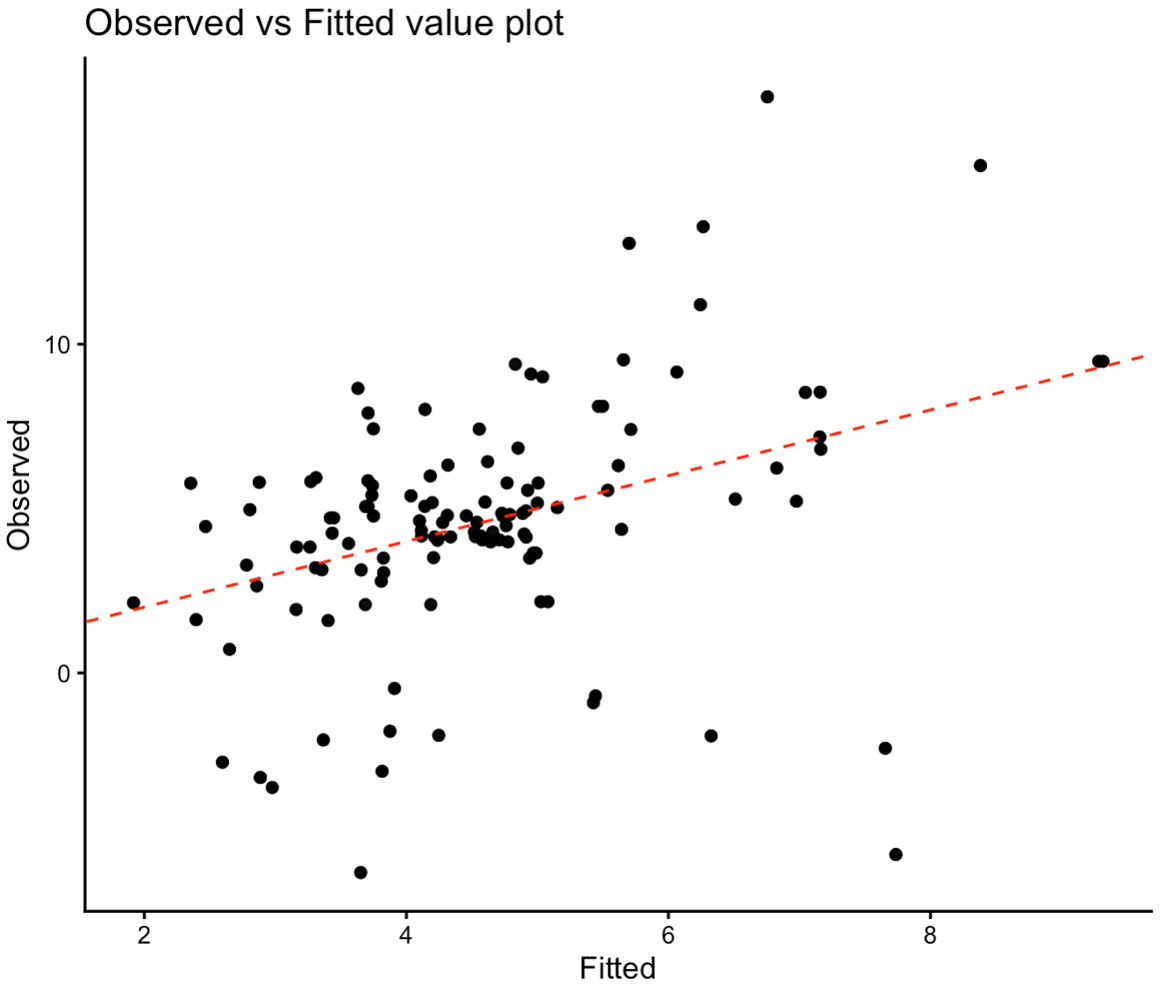


**Figure A8. Model diagnosis result from the Observed vs fitted value plot (midpoint)**


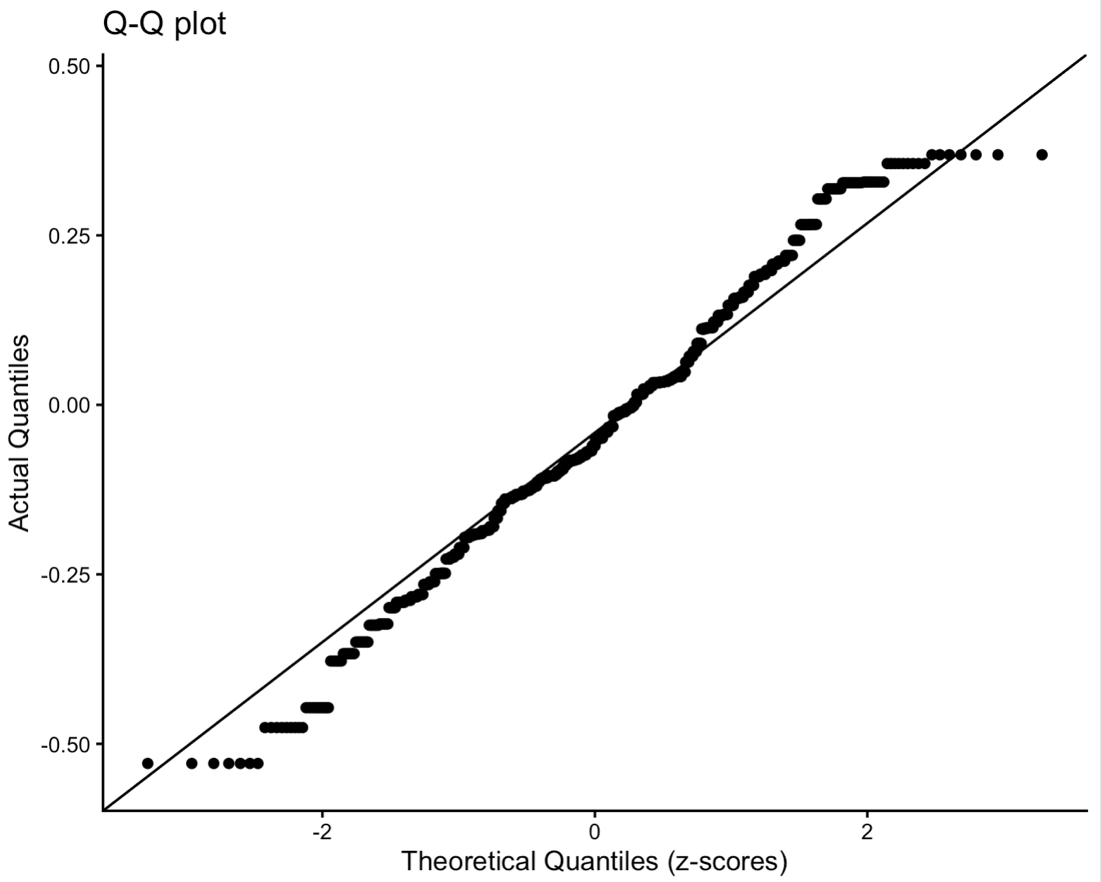


**Figure A9. Model diagnosis result from Q-Q plot (midpoint)**

**Table A1: Model Evaluations general linear mixed-effect model**

|  | RMSE | MAE | MSE |
| --- | --- | --- | --- |
| **Minimum temperature** | | | |
| Temperature sensitivity | 0.02314868 | 0.01626265 | 0.0005367794 |
| Exposure duration | 3.128281 | 2.235021 | 7.211119 |
| Exposure timing (midpoint) | 6.415689 | 2.130625 | 8.252869 |
| **Mean temperature** | | | |
| Temperature sensitivity | 0.02045981 | 0.0142445 | 0.0004196259 |
| Exposure duration | 2.744217 | 2.096904 | 6.291358 |
| Exposure timing (midpoint) | 6.363738 | 2.023031 | 7.505726 |
| **Maximum temperature** | | | |
| Temperature sensitivity | 0.02387835 | 0.01765483 | 0.0005709868 |
| Exposure duration | 3.05658 | 2.268145 | 7.327478 |
| Exposure timing (midpoint) | 6.392129 | 2.043366 | 7.585203 |
