# Supplementary material for: Impact of climate warming on Oncomelania hupensis in China: multi-scale evidence
Source: Infect Dis Poverty. 2026 Jul 3;15:76. doi: 10.1186/s40249-026-01475-0 (PMC13330383; doi:10.1186/s40249-026-01475-0)
Supplement: Supplementary file 2 — Supplementary Material 2. Results from the Cox regression with and without temperature × body size interactions. [file 40249_2026_1475_MOESM2_ESM.docx]

**Table A1. The population benefits from global warming under SSP1-2.6, SSP2-4.5, and SSP5-8.5**

| **Year** | **Scenarios** | **The population benefit from global warming** | ***χ²*** | ***p*** |
| --- | --- | --- | --- | --- |
| **Average** | | | | |
| 2030 | SSP1-2.6 | 246/833 (0.2953181) | 0.0038488 | 0.9981 |
|  | SSP2-4.5 | 245/833 (0.2941176) |  |  |
|  | SSP5-8.5 | 246/833 (0.2953181) |  |  |
| 2050 | SSP1-2.6 | 232/833 (0.2785114) | 0.48669 | 0.784 |
|  | SSP2-4.5 | 232/833 (0.2785114) |  |  |
|  | SSP5-8.5 | 221/833 (0.2653061) |  |  |
| 2070 | SSP1-2.6 | 215/833 (0.2581032) | 3.2828 | 0.1937 |
|  | SSP2-4.5 | 209/833 (0.2509004) |  |  |
|  | SSP5-8.5 | 185/833 (0.2220888) |  |  |
| 2100 | SSP1-2.6 | 205/833 (0.2460984) | 7.8361 | 0.01988* |
|  | SSP2-4.5 | 179/833 (0.214886) |  |  |
|  | SSP5-8.5 | 158/833 (0.1896759) |  |  |
| **CMCC-ESM2** | | | | |
| 2030 | SSP1-2.6 | 258/833 (0.3097239) | 0.23051 | 0.8911 |
|  | SSP2-4.5 | 249/833 (0.2989196) |  |  |
|  | SSP5-8.5 | 254/833 (0.304922) |  |  |
| 2050 | SSP1-2.6 | 226/833 (0.2713085) | 1.6239 | 0.444 |
|  | SSP2-4.5 | 240/833 (0.2881152) |  |  |
|  | SSP5-8.5 | 217/833 (0.2605042) |  |  |
| 2070 | SSP1-2.6 | 216/833 (0.2593037) | 5.7155 | 0.0574 |
|  | SSP2-4.5 | 206/833 (0.2472989) |  |  |
|  | SSP5-8.5 | 176/833 (0.2112845) |  |  |
| 2100 | SSP1-2.6 | 199/833 (0.2388956) | 14.575 | 0.000684*** |
|  | SSP2-4.5 | 183/833 (0.2196879) |  |  |
|  | SSP5-8.5 | 138/833 (0.1656663) |  |  |
| **GFDL-ESM4** | | | | |
| 2030 | SSP1-2.6 | 246/833 (0.2953181) | 0.026836 | 0.9867 |
|  | SSP2-4.5 | 249/833 (0.2989196) |  |  |
|  | SSP5-8.5 | 247/833 (0.2965186) |  |  |
| 2050 | SSP1-2.6 | 230/833 (0.2761104) | 0.22702 | 0.8927 |
|  | SSP2-4.5 | 237/833 (0.2845138) |  |  |
|  | SSP5-8.5 | 229/833 (0.27491) |  |  |
| 2070 | SSP1-2.6 | 216/833 (0.2593037) | 2.607 | 0.2716 |
|  | SSP2-4.5 | 213/833 (0.2557023) |  |  |
|  | SSP5-8.5 | 190/833 (0.2280912) |  |  |
| 2100 | SSP1-2.6 | 205/833 (0.2460984) | 5.3479 | 0.06898 |
|  | SSP2-4.5 | 178/833 (0.2136855) |  |  |
|  | SSP5-8.5 | 167/833 (0.2004802) |  |  |
| **MRI-ESM2-0** | | | | |
| 2030 | SSP1-2.6 | 239/833 (0.2869148) | 0.003915 | 0.998 |
|  | SSP2-4.5 | 238/833 (0.2857143) |  |  |
|  | SSP5-8.5 | 239/833 (0.2869148) |  |  |
| 2050 | SSP1-2.6 | 223/833 (0.2677071) | 0.32374 | 0.8505 |
|  | SSP2-4.5 | 226/833 (0.2713085) |  |  |
|  | SSP5-8.5 | 216/833 (0.2593037) |  |  |
| 2070 | SSP1-2.6 | 211/833 (0.2533013) | 5.2328 | 0.07307 |
|  | SSP2-4.5 | 214/833 (0.2569028) |  |  |
|  | SSP5-8.5 | 178/833 (0.2136855) |  |  |
| 2100 | SSP1-2.6 | 204/833 (0.244898) | 6.4839 | 0.03909* |
|  | SSP2-4.5 | 183/833 (0.2196879) |  |  |
|  | SSP5-8.5 | 161/833 (0.1932773) |  |  |
| **MPI-ESM1-2-HR** | | | | |
| 2030 | SSP1-2.6 | 246/833 (0.2953181) | 0.050193 | 0.9752 |
|  | SSP2-4.5 | 245/833 (0.2941176) |  |  |
|  | SSP5-8.5 | 242/833 (0.2905162) |  |  |
| 2050 | SSP1-2.6 | 231/833 (0.2773109) | 1.3564 | 0.5075 |
|  | SSP2-4.5 | 235/833 (0.2821128) |  |  |
|  | SSP5-8.5 | 215/833 (0.2581032) |  |  |
| 2070 | SSP1-2.6 | 218/833 (0.2617047) | 2.3682 | 0.306 |
|  | SSP2-4.5 | 206/833 (0.2472989) |  |  |
|  | SSP5-8.5 | 191/833 (0.2292917) |  |  |
| 2100 | SSP1-2.6 | 210/833 (0.2521008) | 11.918 | 0.002582** |
|  | SSP2-4.5 | 173/833 (0.2076831) |  |  |
|  | SSP5-8.5 | 153/833 (0.1836735) |  |  |
| **NorESM2-MM** | | | | |
| 2030 | SSP1-2.6 | 244/833 (0.2929172) | 0.015407 | 0.9923 |
|  | SSP2-4.5 | 246/833 (0.2953181) |  |  |
|  | SSP5-8.5 | 246/833 (0.2953181) |  |  |
| 2050 | SSP1-2.6 | 234/833 (0.2809124) | 0.26925 | 0.874 |
|  | SSP2-4.5 | 227/833 (0.272509) |  |  |
|  | SSP5-8.5 | 225/833 (0.270108) |  |  |
| 2070 | SSP1-2.6 | 216/833 (0.2593037) | 3.6986 | 0.1573 |
|  | SSP2-4.5 | 205/833 (0.2460984) |  |  |
|  | SSP5-8.5 | 183/833 (0.2196879) |  |  |
| 2100 | SSP1-2.6 | 204/833 (0.244898) | 3.6827 | 0.1586 |
|  | SSP2-4.5 | 178/833 (0.2136855) |  |  |
|  | SSP5-8.5 | 174/833 (0.2088836) |  |  |
