# Supplementary material for: Impact of climate warming on Oncomelania hupensis in China: multi-scale evidence
Source: Infect Dis Poverty. 2026 Jul 3;15:76. doi: 10.1186/s40249-026-01475-0 (PMC13330383; doi:10.1186/s40249-026-01475-0)
Supplement: Supplementary file 3 — Supplementary Material 3. Comparison of body weight before and after the experiment. [file 40249_2026_1475_MOESM3_ESM.docx]

**Table A1. Distribution of population benefiting from climate warming across the land uses under SSP1-2.6, SSP2-4.5, and SSP5-8.5**

| **Scenarios** | **Land use** | **The population benefit from climate warming** | ***χ²*** | ***p*** |
| --- | --- | --- | --- | --- |
| **Average** | | | | |
| SSP1-2.6 | Crop | 147/205 (0.719528178) | 366.78 | < 2.2e-16*** |
|  | Forest | 35/205 (0.171690695) |  |  |
|  | Grass | 1/205 (0.001310616) |  |  |
|  | Waterbody | 5/205 (0.024901704) |  |  |
|  | Impervious area | 17/205 (0.082568807) |  |  |
| SSP2-4.5 | Crop | 128 (0.719321149) | 319.41 | < 2.2e-16*** |
|  | Forest | 31 (0.171018277) |  |  |
|  | Grass | 1 (0.001305483) |  |  |
|  | Waterbody | 4 (0.024804178) |  |  |
|  | Impervious area | 15 (0.083550914) |  |  |
| SSP5-8.5 | Crop | 113 (0.719736842) | 282.19 | < 2.2e-16*** |
|  | Forest | 27 (0.171052632) |  |  |
|  | Grass | 1 (0.001315789) |  |  |
|  | Waterbody | 4 (0.025000000) |  |  |
|  | Impervious area | 13 (0.082894737) |  |  |
| **CMCC-ESM2** | | | | |
| SSP1-2.6 | Crop | 156/218 (0.719528178) | 390.53 | < 2.2e-16*** |
|  | Forest | 37 (0.171690695) |  |  |
|  | Grass | 1 (0.001310616) |  |  |
|  | Waterbody | 5 (0.024901704) |  |  |
|  | Impervious area | 18 (0.082568807) |  |  |
| SSP2-4.5 | Crop | 141 (0.719321149) | 348.31 | < 2.2e-16*** |
|  | Forest | 34 (0.171018277) |  |  |
|  | Grass | 1 (0.001305483) |  |  |
|  | Waterbody | 5/198 (0.024804178) |  |  |
|  | Impervious area | 17 (0.083550914) |  |  |
| SSP5-8.5 | Crop | 107 (0.719736842) | 266.67 | < 2.2e-16*** |
|  | Forest | 26 (0.171052632) |  |  |
|  | Grass | 1 (0.001315789) |  |  |
|  | Waterbody | 4/150 (0.025000000) |  |  |
|  | Impervious area | 12 (0.082894737) |  |  |
| **GFDL-ESM4** | | | | |
| SSP1-2.6 | Crop | 161 (0.720472441) | 399.11 | < 2.2e-16*** |
|  | Forest | 38 (0.170603675) |  |  |
|  | Grass | 1 (0.001312336) |  |  |
|  | Waterbody | 6/225 (0.024934383) |  |  |
|  | Impervious area | 19 (0.082677165) |  |  |
| SSP2-4.5 | Crop | 140 (0.72026144) | 346.46 | < 2.2e-16*** |
|  | Forest | 33 (0.16993464) |  |  |
|  | Grass | 1 (0.00130719) |  |  |
|  | Waterbody | 5/195 (0.02483660) |  |  |
|  | Impervious area | 16 (0.08366013) |  |  |
| SSP5-8.5 | Crop | 133 (0.720685112) | 329.59 | < 2.2e-16*** |
|  | Forest | 31 (0.169960474) |  |  |
|  | Grass | 1 (0.001317523) |  |  |
|  | Waterbody | 5 (0.025032938) |  |  |
|  | Impervious area | 14/184 (0.083003953) |  |  |
| **MPI-ESM1-2-HR** | | | | |
| SSP1-2.6 | Crop | 166/232 (0.719160105) | 412.09 | < 2.2e-16*** |
|  | Forest | 40 (0.171916010) |  |  |
|  | Grass | 1 (0.001312336) |  |  |
|  | Waterbody | 6 (0.024934383) |  |  |
|  | Impervious area | 19 (0.082677165) |  |  |
| SSP2-4.5 | Crop | 133/187 (0.71895425) | 328 | < 2.2e-16*** |
|  | Forest | 32 (0.17124183) |  |  |
|  | Grass | 1 (0.00130719) |  |  |
|  | Waterbody | 5 (0.02483660) |  |  |
|  | Impervious area | 16 (0.08366013) |  |  |
| SSP5-8.5 | Crop | 120/167 (0.719367589) | 295.66 | < 2.2e-16*** |
|  | Forest | 29 (0.171277997) |  |  |
|  | Grass | 1 (0.001317523) |  |  |
|  | Waterbody | 4 (0.025032938) |  |  |
|  | Impervious area | 13 (0.083003953) |  |  |
| **MRI-ESM2-0** | | | | |
| SSP1-2.6 | Crop | 160/224 (0.71952817) | 398.64 | < 2.2e-16*** |
|  | Forest | 38 (0.171690695) |  |  |
|  | Grass | 1 (0.001310616) |  |  |
|  | Waterbody | 6 (0.024901704) |  |  |
|  | Impervious area | 18 (0.082568807) |  |  |
| SSP2-4.5 | Crop | 144/201 (0.719321149) | 372.05 | < 2.2e-16*** |
|  | Forest | 34 (0.171018277) |  |  |
|  | Grass | 1 (0.001305483) |  |  |
|  | Waterbody | 5 (0.024804178) |  |  |
|  | Impervious area | 17 (0.083550914) |  |  |
| SSP5-8.5 | Crop | 127 (0.719736842) | 318.06 | < 2.2e-16*** |
|  | Forest | 30 (0.171052632) |  |  |
|  | Grass | 1 (0.001315789) |  |  |
|  | Waterbody | 4/178 (0.025000000) |  |  |
|  | Impervious area | 15 (0.082894737) |  |  |
| **NorESM2-MM** | | | | |
| SSP1-2.6 | Crop | 146 (0.719528178) | 363.35 | < 2.2e-16*** |
|  | Forest | 35 (0.171690695) |  |  |
|  | Grass | 1 (0.001310616) |  |  |
|  | Waterbody | 5/204 (0.024901704) |  |  |
|  | Impervious area | 17 (0.082568807) |  |  |
| SSP2-4.5 | Crop | 127/178 (0.719321149) | 318.06 | < 2.2e-16*** |
|  | Forest | 30 (0.171018277) |  |  |
|  | Grass | 1 (0.001305483) |  |  |
|  | Waterbody | 4 (0.024804178) |  |  |
|  | Impervious area | 15 (0.083550914) |  |  |
| SSP5-8.5 | Crop | 124/174 (0.719736842) | 310.73 | < 2.2e-16*** |
|  | Forest | 30 (0.171052632) |  |  |
|  | Grass | 1 (0.001315789) |  |  |
|  | Waterbody | 4 (0.025000000) |  |  |
|  | Impervious area | 14 (0.082894737) |  |  |
