# Supplementary material for: Impact of climate warming on Oncomelania hupensis in China: multi-scale evidence
Source: Infect Dis Poverty. 2026 Jul 3;15:76. doi: 10.1186/s40249-026-01475-0 (PMC13330383; doi:10.1186/s40249-026-01475-0)
Supplement: Supplementary file 4 — Supplementary Material 4. Relationship between the midpoint and sensitivity (results from minimum and maximum temperature). [file 40249_2026_1475_MOESM4_ESM.docx]

**Table A1. Predicted density across land uses under SSP1-2.6, SSP2-4.5, SSP5-8.5**

| **Scenarios** | **Land use** | **Predicted density**  **medium (IQR)** | ***χ²*** | ***p*** |
| --- | --- | --- | --- | --- |
| **Average** | | | | |
| SSP1-2.6 | Crop | 0.0943 (0/0.228) | 73.507 | 4.121e-15 *** |
|  | Forest | 0.0912 (0/0.209) |  |  |
|  | Grass | 0.0544 (0/0.169) |  |  |
|  | Waterbody | 0.113 (0.093/0.255) |  |  |
|  | Impervious area | 0.0421 (0/0.0605) |  |  |
| SSP2-4.5 | Crop | 0.0623 (0/0.187) | 47.747 | 1.066e-09 *** |
|  | Forest | 0.0579 (0/0.186) |  |  |
|  | Grass | 0.0471 (0/0.157) |  |  |
|  | Waterbody | 0.0796 (0.012/0.175) |  |  |
|  | Impervious area | 0.0184 (0/0.178) |  |  |
| SSP5-8.5 | Crop | 0.0586 (0/0.153) | 66.17 | 1.459e-13 *** |
|  | Forest | 0.0532 (0/0.167) |  |  |
|  | Grass | 0.0319 (0/0.136) |  |  |
|  | Waterbody | 0.0730 (0.001/0.169) |  |  |
|  | Impervious area | 0.0222 (0/0.139) |  |  |
| **CMCC-ESM2** | | | | |
| SSP1-2.6 | Crop | 0.0771 (0/0.207) | 77.957 | 4.718e-16 *** |
|  | Forest | 0.0615 (0/0.196) |  |  |
|  | Grass | 0.0629 (0/0.173) |  |  |
|  | Waterbody | 0.105 (0.058/0.232) |  |  |
|  | Impervious area | 0.0522 (0/0.085) |  |  |
| SSP2-4.5 | Crop | 0.0650 (0/0.193) | 59.878 | 3.077e-12 *** |
|  | Forest | 0.0597 (0/0.190) |  |  |
|  | Grass | 0.0469 (0/0.176) |  |  |
|  | Waterbody | 0.0838 (0.002/0.183) |  |  |
|  | Impervious area | 0.0286 (0/0.150) |  |  |
| SSP5-8.5 | Crop | 0.0483 (0/0.129) | 75.215 | 1.794e-15 *** |
|  | Forest | 0.0460 (0/0.145) |  |  |
|  | Grass | 0.0310 (0/-0.210) |  |  |
|  | Waterbody | 0.0595 (0.000/0.137) |  |  |
|  | Impervious area | 0.0221 (0/0.119) |  |  |
| **GFDL-ESM4** | | | | |
| SSP1-2.6 | Crop | 0.0829 (0/0.238) | 95.811 | < 2.2e-16 *** |
|  | Forest | 0.0649 (0/0.212) |  |  |
|  | Grass | 0.0486 (0/0.170) |  |  |
|  | Waterbody | 0.116 (0.045/0.263) |  |  |
|  | Impervious area | 0.0350 (0/0.042) |  |  |
| SSP2-4.5 | Crop | 0.0614 (0/0.185) | 65.159 | 2.382e-13 *** |
|  | Forest | 0.0575 (0/0.188) |  |  |
|  | Grass | 0.0416 (0/0.147) |  |  |
|  | Waterbody | 0.0786 (0.000/0.173) |  |  |
|  | Impervious area | 0.0180 (0/0.174) |  |  |
| SSP5-8.5 | Crop | 0.0627 (0/0.165) | 72.046 | 8.391e-15 *** |
|  | Forest | 0.0561 (0/0.176) |  |  |
|  | Grass | 0.0342 (0/0.135) |  |  |
|  | Waterbody | 0.0775 (0.010/0.182) |  |  |
|  | Impervious area | 0.0214 (0/0.148) |  |  |
| **MPI-ESM1-2-HR** | | | | |
| SSP1-2.6 | Crop | 0.0868 (0/0.237) | 100.05 | < 2.2e-16 *** |
|  | Forest | 0.0637 (0/0.213) |  |  |
|  | Grass | 0.0538 (0/0.167) |  |  |
|  | Waterbody | 0.120 (0.027/0.267) |  |  |
|  | Impervious area | 0.0510 (0/0.213) |  |  |
| SSP2-4.5 | Crop | 0.0590 (0/0.174) | 53.394 | 7.049e-11 *** |
|  | Forest | 0.0543 (0/0.174) |  |  |
|  | Grass | 0.0450 (0/0.144) |  |  |
|  | Waterbody | 0.0740 (0.0310/0.165) |  |  |
|  | Impervious area | 0.0190 (0/0.167) |  |  |
| SSP5-8.5 | Crop | 0.0556 (0/0.141) | 82.386 | < 2.2e-16 *** |
|  | Forest | 0.0506 (0/0.157) |  |  |
|  | Grass | 0.0317 (0/0.135) |  |  |
|  | Waterbody | 0.0691 (0.000/0.158) |  |  |
|  | Impervious area | 0.0227 (0/0.132) |  |  |
| **MRI-ESM2-0** | | | | |
| SSP1-2.6 | Crop | 0.0837 (0/0.234) | 33.34 | 1.017e-06 *** |
|  | Forest | 0.0649 (0/0.216) |  |  |
|  | Grass | 0.0533 (0/0.165) |  |  |
|  | Waterbody | 0.114 (0.020/0.261) |  |  |
|  | Impervious area | 0.0315 (0/0.165) |  |  |
| SSP2-4.5 | Crop | 0.0655 (0/0.191) | 54.392 | 4.355e-11 |
|  | Forest | 0.0607 (0/0.194) |  |  |
|  | Grass | 0.0454 (0/0.158) |  |  |
|  | Waterbody | 0.0813 (0.000/0.178) |  |  |
|  | Impervious area | 0.0206 (0/0.183) |  |  |
| SSP5-8.5 | Crop | 0.0608 (0/0.157) | 74.386 | 2.687e-15 *** |
|  | Forest | 0.0552 (0/0.173) |  |  |
|  | Grass | 0.0309 (0/0.133) |  |  |
|  | Waterbody | 0.0760 (0.011/0.177) |  |  |
|  | Impervious area | 0.0216 (0/0.143) |  |  |
| **NorESM2-MM** | | | | |
| SSP1-2.6 | Crop | 0.0818 (0/0.227) | 88.425 | < 2.2e-16 *** |
|  | Forest | 0.0632 (0/0.207) |  |  |
|  | Grass | 0.0554 (0/0.171) |  |  |
|  | Waterbody | 0.115 (0.060/0.255) |  |  |
|  | Impervious area | 0.0323 (0/0.208) |  |  |
| SSP2-4.5 | Crop | 0.0610 (0/0.190) | 63.92 | 4.345e-13 *** |
|  | Forest | 0.0570 (0/0.187) |  |  |
|  | Grass | -0.0568 (0/0.159) |  |  |
|  | Waterbody | 0.0803 (0.001/0.177) |  |  |
|  | Impervious area | 0.0120 (0/0.182) |  |  |
| SSP5-8.5 | Crop | 0.0658 (0/0.172) | 84.267 | < 2.2e-16 *** |
|  | Forest | 0.0580 (0/0.185) |  |  |
|  | Grass | 0.0315 (0/0.130) |  |  |
|  | Waterbody | 0.0826 (0.0173/0.191) |  |  |
|  | Impervious area | 0.0233 (0/0.154) |  |  |

**Table A2. Result from Pairwise Wilcoxon rank-sum tests**

| **Model and scenarios** | **Land use** | **Waterbody** | **Crop** | **Forest** | **Grass** |
| --- | --- | --- | --- | --- | --- |
| Average  SSP1-2.6 | Crop | 0.00065 *** | - | - | - |
|  | Forest | 5.0e-07 *** | 2.6e-09*** | - | - |
|  | Grass | 0.000111*** | 0.00011004*** | 0.0010004** | - |
|  | Impervious area | 8.3e-06 *** | 3.7e-06*** | 0.000259*** | 0.000259*** |
| Average  SSP2-4.5 | Crop | 0.00024 *** | - | - | - |
|  | Forest | 9.3e-05 *** | 0.00026*** | - | - |
|  | Grass | 0.0031** | 0.00211** | 0.0011** | - |
|  | Impervious area | 4.3e-05 *** | 5.2e-06*** | 8.3e-05*** | 0.000127*** |
| Average  SSP5-8.5 | Crop | 0.000293 *** | - | - | - |
|  | Forest | 0.00018 *** | 3.2e-06*** | - | - |
|  | Grass | 0.00438 ** | 0.00104** | 0.00143** | - |
|  | Impervious area | 2.2e-05 *** | 1.4e-07*** | 0.00021*** | 0.0001048*** |
| CMCC-ESM2  SSP1-2.6 | Crop | 0.00048*** | - | - | - |
|  | Forest | 2.6e-06 *** | 2.9e-08*** | - | - |
|  | Grass | 0.00101** | 0.0010300** | 0.0018000** | - |
|  | Impervious area | 8.3e-06 *** | 1.6e-07*** | 0.00013*** | 0. 0001000*** |
| CMCC-ESM2  SSP2-4.5 | Crop | 0.000219 *** | - | - | - |
|  | Forest | 4.5e-05 *** | 0.00027*** | - | - |
|  | Grass | 0.001771** | 0.0013611** | 0.0011351** | - |
|  | Impervious area | 1.3e-05 *** | 1.4e-07 *** | 1.3e-05*** | 0.0001247*** |
| CMCC-ESM2  SSP5-8.5 | Crop | 0.00080*** | - | - | - |
|  | Forest | 9.3e-05 *** | 7.6e-07 *** | - | - |
|  | Grass | 0.001300** | 0.00100670** | 0.001350** | - |
|  | Impervious area | 7.4e-06 *** | 1.1e-08 *** | 0.00011*** | 0. 000100*** |
| GFDL-ESM4  SSP1-2.6 | Crop | 0.00061 *** | - | - | - |
|  | Forest | 1.5e-07 *** | 3.1e-11*** | - | - |
|  | Grass | 0.0013111** | 0.0025611** | 0.00351** | - |
|  | Impervious area | 1.4e-06 *** | 5.6e-09*** | 0.0022*** | 0.0001675*** |
| GFDL-ESM4  SSP2-4.5 | Crop | 0.000150*** | - | - | - |
|  | Forest | 3.8e-05 *** | 0. 00018*** | - | - |
|  | Grass | 0.00353111** | 0.00658111** | 0.00176211** | - |
|  | Impervious area | 7.9e-06 *** | 1.4e-08*** | 7.9e-06*** | 0.0001511*** |
| GFDL-ESM4  SSP5-8.5 | Crop | 0.000508*** | - | - | - |
|  | Forest | 0.00023 *** | 1.5e-06*** | - | - |
|  | Grass | 0.00145411** | 0.0011871** | 0.0011123** | - |
|  | Impervious area | 1.5e-05 *** | 6.5e-09*** | 9.3e-05*** | 0.00011374*** |
| MPI-ESM1-2-HR  SSP1-2.6 | Crop | 0.00044 *** | - | - | - |
|  | Forest | 7.8e-08 *** | 2.2e-12*** | - | - |
|  | Grass | 0.00111** | 0.0010974** | 0.0010974** | - |
|  | Impervious area | 1.3e-06 *** | 9.0e-09*** | 0.00030*** | 0.0002004*** |
| MPI-ESM1-2-HR  SSP2-4.5 | Crop | 0.000505*** | - | - | - |
|  | Forest | 0.00042 *** | 0.000505*** | - | - |
|  | Grass | 0. 003400** | 0. 0017200** | 0. 001040** | - |
|  | Impervious area | 1.7e-05*** | 1.3e-07*** | 1.0e-05*** | 0.0001000*** |
| MPI-ESM1-2-HR  SSP5-8.5 | Crop | 0.000262*** | - | - | - |
|  | Forest | 8.7e-05 *** | 1.2e-08*** | - | - |
|  | Grass | 0.0010035** | 0.0035000** | 0.0017500** | - |
|  | Impervious area | 1.8e-05 *** | 2.8e-09*** | 0.00015*** | 0.0001000*** |
| MRI-ESM2-0  SSP1-2.6 | Crop | 0.9548 | - | - | - |
|  | Forest | 0.9287 | 0.9548 | - | - |
|  | Grass | 0.02121* | 0.01119* | 0.0224* | - |
|  | Impervious area | 0.00035 *** | 8.6e-07*** | 9.3e-07*** | 0.0004710*** |
| MRI-ESM2-0  SSP2-4.5 | Crop | 0.000253*** | - | - | - |
|  | Forest | 0.00026*** | 0.000253*** | - | - |
|  | Grass | 0.003511 ** | 0.0011121** | 0.00175511** | - |
|  | Impervious area | 1.8e-05 *** | 2.5e-07*** | 1.3e-05*** | 0.0001240*** |
| MRI-ESM2-0  SSP5-8.5 | Crop | 0.00347*** | - | - | - |
|  | Forest | 0.00013 *** | 6.1e-07*** | - | - |
|  | Grass | 0.0014462 ** | 0.00115422** | 0.0002252*** | - |
|  | Impervious area | 2.1e-05 *** | 5.4e-09*** | 9.7e-05*** | 0.0001020*** |
| NorESM2-MM  SSP1-2.6 | Crop | 0.00036*** | - | - | - |
|  | Forest | 2.1e-07 *** | 5.3e-11*** | - | - |
|  | Grass | 0.0046111** | 0.00013511*** | 0.0003651*** | - |
|  | Impervious area | 2.9e-06 *** | 2.1e-07*** | 0.00031*** | 0.0001830*** |
| NorESM2-MM  SSP2-4.5 | Crop | 0.00066 *** | - | - | - |
|  | Forest | 2.6e-05 *** | 0.00021*** | - | - |
|  | Grass | 0.001140** | 0.00190** | 0. 001160** | - |
|  | Impervious area | 6.9e-06 *** | 5.4e-08*** | 1.0e-05*** | 0.0001000*** |
| NorESM2-MM  SSP5-8.5 | Crop | 0.000207 *** | - | - | - |
|  | Forest | 3.5e-05 *** | 5.6e-09*** | - | - |
|  | Grass | 0.003511** | 0.0071** | 0.001461** | - |
|  | Impervious area | 4.7e-06 *** | 3.4e-09*** | 0.00016*** | 0.0001130*** |
