# Supplementary material for: Impact of climate warming on Oncomelania hupensis in China: multi-scale evidence
Source: Infect Dis Poverty. 2026 Jul 3;15:76. doi: 10.1186/s40249-026-01475-0 (PMC13330383; doi:10.1186/s40249-026-01475-0)
Supplement: Supplementary file 5 — Supplementary Material 5. Result from general mixed function (maximum temperature). [file 40249_2026_1475_MOESM5_ESM.docx]

**Table A1. Predicted growth rate across land uses in GCMs**

| **Scenarios** | **Land use** | **Growth rate**  **(IQR)** | ***χ²*** | ***p*** |
| --- | --- | --- | --- | --- |
| **Average** | | | | |
| SSP1-2.6 | Crop | 1.36 (-1.91/17.6) | 33.627 | 8.888e-07 *** |
|  | Forest | 2.44 (-0.943/14.6) |  |  |
|  | Grass | -1.739 (-3.250/1.730) |  |  |
|  | Waterbody | -0.0115 (-0.894/5.13) |  |  |
|  | Impervious area | -1.065 (-3.86/1.93) |  |  |
| SSP2-4.5 | Crop | 0.987 (-2.11/15.6) | 23.621 | 9.514e-05 *** |
|  | Forest | 2.07 (-1.21/13.5) |  |  |
|  | Grass | -1.635 (-3.318/3.315) |  |  |
|  | Waterbody | -0.265 (-1.01/3.55) |  |  |
|  | Impervious area | -0.439 (-3.32/2.35) |  |  |
| SSP5-8.5 | Crop | 0.588 (-1.34/11.0) | 37.088 | 1.727e-07 *** |
|  | Forest | 1.45 (-1.32/11.8) |  |  |
|  | Grass | -1.429 (-3.470/1.478) |  |  |
|  | Waterbody | -0.336 (-0.993/3.18) |  |  |
|  | Impervious area | -1.175 (-3.47/1.39) |  |  |
| **CMCC-ESM2** | | | | |
| SSP1-2.6 | Crop | 1.20 (-1.83/16.9) | 33.92 | 7.737e-07 *** |
|  | Forest | 2.36 (-0.946/14.3) |  |  |
|  | Grass | -1.85 (-3.45/1.53) |  |  |
|  | Waterbody | -0.108 (-0.901/4.44) |  |  |
|  | Impervious area | -1.06 (-3.65/1.92) |  |  |
| SSP2-4.5 | Crop | 1.09 (-2.17/16.5) | 23.576 | 9.711e-05 *** |
|  | Forest | 2.21 (-1.21/14.5) |  |  |
|  | Grass | -1.63 (-3.27/1.70) |  |  |
|  | Waterbody | -0.222 (-1.01/3.84) |  |  |
|  | Impervious area | -0.387 (-3.47/2.50) |  |  |
| SSP5-8.5 | Crop | 0.333 (-1.15/9.59) | 34.502 | 5.879e-07 *** |
|  | Forest | 1.18 (-1.00/10.8) |  |  |
|  | Grass | -1.42 (-3.12/1.62) |  |  |
|  | Waterbody | -0.451 (-0.980/2.47) |  |  |
|  | Impervious area | -1.07 (-2.62/1.28) |  |  |
| **GFDL-ESM4** | | | | |
| SSP1-2.6 | Crop | 1.37 (-1.90/17.5) | 33.732 | 8.459e-07 *** |
|  | Forest | 2.35 (-0.940/15.1) |  |  |
|  | Grass | -1.66 (-3.56/1.71) |  |  |
|  | Waterbody | 0.00932 (-0.894/5.29) |  |  |
|  | Impervious area | -1.05 (-3.73/1.88) |  |  |
| SSP2-4.5 | Crop | 0.929 (-2.02/15.4) | 23.677 | 9.271e-05 *** |
|  | Forest | 1.90 (-1.21/13.4) |  |  |
|  | Grass | -1.56 (-3.44/1.46) |  |  |
|  | Waterbody | -0.261 (-1.01/3.64) |  |  |
|  | Impervious area | -0.467 (-3.30/2.18) |  |  |
| SSP5-8.5 | Crop | 0.716 (-1.43/11.9) | 36.752 | 2.026e-07 *** |
|  | Forest | 1.63 (-1.57/12.2) |  |  |
|  | Grass | -1.46 (-3.46/1.16) |  |  |
|  | Waterbody | -0.312 (-0.997/3.25) |  |  |
|  | Impervious area | -1.29 (-4.14/1.58) |  |  |
| **MPI-ESM1-2-HR** | | | | |
| SSP1-2.6 | Crop | 1.47 (-1.95/19.6) | 33.205 | 1.085e-06 *** |
|  | Forest | 2.57 (-0.945/14.2) |  |  |
|  | Grass | -1.72 (-3.82/1.62) |  |  |
|  | Waterbody | 0.0450 (-0.888/5.52) |  |  |
|  | Impervious area | -1.07 (-4.23/2.01) |  |  |
| SSP2-4.5 | Crop | 0.853 (-1.82/14.5) | 23.588 | 9.66e-05 *** |
|  | Forest | 1.98 (-1.16/12.2) |  |  |
|  | Grass | -1.61 (-3.16/1.41) |  |  |
|  | Waterbody | -0.326 (-1.00/3.14) |  |  |
|  | Impervious area | -0.427 (-3.00/2.17) |  |  |
| SSP5-8.5 | Crop | 0.496 (-1.17/9.47) | 36.292 | 2.52e-07 *** |
|  | Forest | 1.26 (-1.08/11.3) |  |  |
|  | Grass | -1.43 (-3.34/1.47) |  |  |
|  | Waterbody | -0.361 (-0.993/3.06) |  |  |
|  | Impervious area | -1.07 (-2.88/1.40) |  |  |
| **MRI-ESM2-0** | | | | |
| SSP1-2.6 | Crop | 1.42 (-1.87/18.7) | 33.424 | 9.778e-07 *** |
|  | Forest | 2.56 (-0.940/15.3) |  |  |
|  | Grass | -1.72 (-3.82/1.87) |  |  |
|  | Waterbody | -0.00356 (-0.894/5.22) |  |  |
|  | Impervious area | -1.04 (-3.61/1.98) |  |  |
| SSP2-4.5 | Crop | 1.09 (-2.02/18.0) | 23.538 | 9.882e-05 *** |
|  | Forest | 2.20 (-1.20/13.8) |  |  |
|  | Grass | -1.61 (-3.11/1.31) |  |  |
|  | Waterbody | -0.256 (-1.01/3.59) |  |  |
|  | Impervious area | -0.398 (-3.06/2.56) |  |  |
| SSP5-8.5 | Crop | 0.641 (-1.41/12.0) | 37.095 | 1.722e-07 *** |
|  | Forest | 1.49 (-1.47/11.5) |  |  |
|  | Grass | -1.42 (-3.52/1.22) |  |  |
|  | Waterbody | -0.302 (-1.00/3.42) |  |  |
|  | Impervious area | -1.20 (-3.82/1.32) |  |  |
| **NorESM2-MM** | | | | |
| SSP1-2.6 | Crop | 1.32 (-1.93/17.0) | 33.882 | 7.88e-07 *** |
|  | Forest | 2.38 (-0.943/14.2) |  |  |
|  | Grass | -1.75 (-3.85 /1.55) |  |  |
|  | Waterbody | -0.0000686 (-0.895/5.19) |  |  |
|  | Impervious area | -1.10 (-4.09/1.85) |  |  |
| SSP2-4.5 | Crop | 0.961 (-2.46/16.0) | 23.455 | 0.0001027 *** |
|  | Forest | 2.07 (-1.26/13.6) |  |  |
|  | Grass | -1.77 (-3.77/1.9104) |  |  |
|  | Waterbody | -0.263 (-1.02/3.54) |  |  |
|  | Impervious area | -0.518 (-3.81/2.32) |  |  |
| SSP5-8.5 | Crop | 0.772 (-1.46/11.8) | 36.919 | 1.872e-07 *** |
|  | Forest | 1.68 (-1.43/12.8) |  |  |
|  | Grass | -1.42 (-3.12/1.52) |  |  |
|  | Waterbody | -0.253 (-0.996/3.68) |  |  |
|  | Impervious area | -1.26 (-3.97/1.41) |  |  |

**Table A2. Result from Pairwise Wilcoxon rank-sum tests**

| **Model and scenarios** | **Land use** | **Waterbody** | **Crop** | **Forest** | **Grass** |
| --- | --- | --- | --- | --- | --- |
| Average  SSP1-2.6 | Crop | 0.9365 | - | - | - |
|  | Forest | 0.8602 | 0.9365 | - | - |
|  | Grass | 0.002150** | 0.00316** | 0.002073** | - |
|  | Impervious areas | 0.0031** | 7.3e-07*** | 8.9e-07*** | 0.4710 |
| Average  SSP2-4.5 | Crop | 0.65533 | - | - | - |
|  | Forest | 0.73449 | 0.42714 | - | - |
|  | Grass | 0.002097 ** | 0.003267** | 0.002307** | - |
|  | Impervious areas | 0.01082 * | 0.00015** | 3.1e-05*** | 0.39198 |
| Average  SSP5-8.5 | Crop | 0. 8088 | - | - | - |
|  | Forest | 0. 8088 | 0.7003 | - | - |
|  | Grass | 0.00250 ** | 0.0050** | 0.0010** | - |
|  | Impervious areas | 0.0039 ** | 6.7e-08*** | 3.2e-06 *** | 0.3336 |
| CMCC-ESM2  SSP1-2.6 | Crop | 0.9297 | - | - | - |
|  | Forest | 0.8714 | 0.8714 | - | - |
|  | Grass | 0.001996** | 0.001637** | 0. 002746** | - |
|  | Impervious areas | 0.0032 ** | 6.8e-07 *** | 6.8e-07 *** | 0.3983 |
| CMCC-ESM2  SSP2-4.5 | Crop | 0.64236 | - | - | - |
|  | Forest | 0.71753 | 0.44138 | - | - |
|  | Grass | 0.00220 ** | 0.0280 * | 0.020* | - |
|  | Impervious areas | 0.01009 * | 0.00016** | 3.3e-05*** | 0. 39198 |
| CMCC-ESM2  SSP5-8.5 | Crop | 0.9319 | - | - | - |
|  | Forest | 0.9319 | 0.9319 | - | - |
|  | Grass | 0.02136 * | 0.236 | 0.134 | - |
|  | Impervious areas | 0.0058 ** | 3.4e-07 *** | 1.8e-06 *** | 0.3336 |
| GFDL-ESM4 SSP1-2.6 | Crop | 0.9119 | - | - | - |
|  | Forest | 0.9119 | 0.9119 | - | - |
|  | Grass | 0.0258 * | 0.0128 * | 0.2258 | - |
|  | Impervious areas | 0.0031 ** | 6.8e-07 *** | 8.1e-07 *** | 0.5104 |
| GFDL-ESM4  SSP2-4.5 | Crop | 0. 63913 | - | - | - |
|  | Forest | 0.72413 | 0.43896 | - | - |
|  | Grass | 0.0230 ** | 0.0097 ** | 0.634 | - |
|  | Impervious areas | 0.01121 * | 0.00014 ** | 3.5e-05 *** | 0.43896 |
| GFDL-ESM4  SSP5-8.5 | Crop | 0.8116 | - | - | - |
|  | Forest | 0.8116 | 0.5469 | - | - |
|  | Grass | 0.029 ** | 0.2459 | 0.2500 | - |
|  | Impervious areas | 0.0037 ** | 7.8e-08 *** | 5.9e-06 *** | 0.3983 |
| MPI-ESM1-2-HR  SSP1-2.6 | Crop | 0.917 | - | - | - |
|  | Forest | 0.640 | 0.893 | - | - |
|  | Grass | 0.0021** | 0.0018** | 0.00113** | - |
|  | Impervious areas | 0.003 ** | 8.0e-07 *** | 1.8e-06 *** | 0.471 |
| MPI-ESM1-2-HR  SSP2-4.5 | Crop | 0.63034 | - | - | - |
|  | Forest | 0.75588 | 0.42788 | - | - |
|  | Grass | 0.00243 ** | 0.00419** | 0.0518 | - |
|  | Impervious areas | 0.01244 * | 0.00016** | 3.1e-05 *** | 0.35951 |
| MPI-ESM1-2-HR  SSP5-8.5 | Crop | 0.869 | - | - | - |
|  | Forest | 0.896 | 0.869 | - | - |
|  | Grass | 0.002** | 0.0014** | 0.00736** | - |
|  | Impervious areas | 0.006 ** | 1.1e-07 *** | 2.5e-06 *** | 0.334 |
| MRI-ESM2-0  SSP1-2.6 | Crop | 0.9762 | - | - | - |
|  | Forest | 0.9287 | 0.9762 | - | - |
|  | Grass | 0.00224 ** | 0.0012 ** | 0.00563** | - |
|  | Impervious areas | 0.0035 ** | 8.10-07 *** | 9.3e-07 *** | 0.4710 |
| MRI-ESM2-0  SSP2-4.5 | Crop | 0. 66186 | - | - | - |
|  | Forest | 0.77746 | 0.41198 | - | - |
|  | Grass | 0.00820** | 0.0020** | 0.00220** | - |
|  | Impervious areas | 0.01244 * | 0.00017 ** | 2.9e-05 *** | 0. 39198 |
| MRI-ESM2-0  SSP5-8.5 | Crop | 0.8309 | - | - | - |
|  | Forest | 0.8309 | 0.7062 | - | - |
|  | Grass | 0.00459 ** | 0.00255 ** | 0.00289** | - |
|  | Impervious areas | 0.0039 ** | 6.5e-08 *** | 3.2e-06 *** | 0.3649 |
| NorESM2-MM  SSP1-2.6 | Crop | 0.9275 | - | - | - |
|  | Forest | 0.8863 | 0.9275 | - | - |
|  | Grass | 0.00150 ** | 0.00240 ** | 0.00210** | - |
|  | Impervious areas | 0.0031 ** | 6.8e-07 *** | 7.0e-07 *** | 0.4710 |
| NorESM2-MM  SSP2-4.5 | Crop | 0.63057 | - | - | - |
|  | Forest | 0.68818 | 0.47509 | - | - |
|  | Grass | 0.0023377 ** | 0.0023377 ** | 0.0023377** | - |
|  | Impervious areas | 0.01045 * | 0.00016** | 3.1e-05 *** | 0.42640 |
| NorESM2-MM  SSP5-8.5 | Crop | 0.8172 | - | - | - |
|  | Forest | 0.8069 | 0.5612 | - | - |
|  | Grass | 0.00267 ** | 0.00736 ** | 0.00156** | - |
|  | Impervious areas | 0.0032 ** | 7.7e-08 *** | 4.2e-06 *** | 0.4336 |
