# Supplementary figures and images for: Impact of climate warming on Oncomelania hupensis in China: multi-scale evidence
Source: Infect Dis Poverty. 2026 Jul 3;15:76. doi: 10.1186/s40249-026-01475-0 (PMC13330383; doi:10.1186/s40249-026-01475-0)

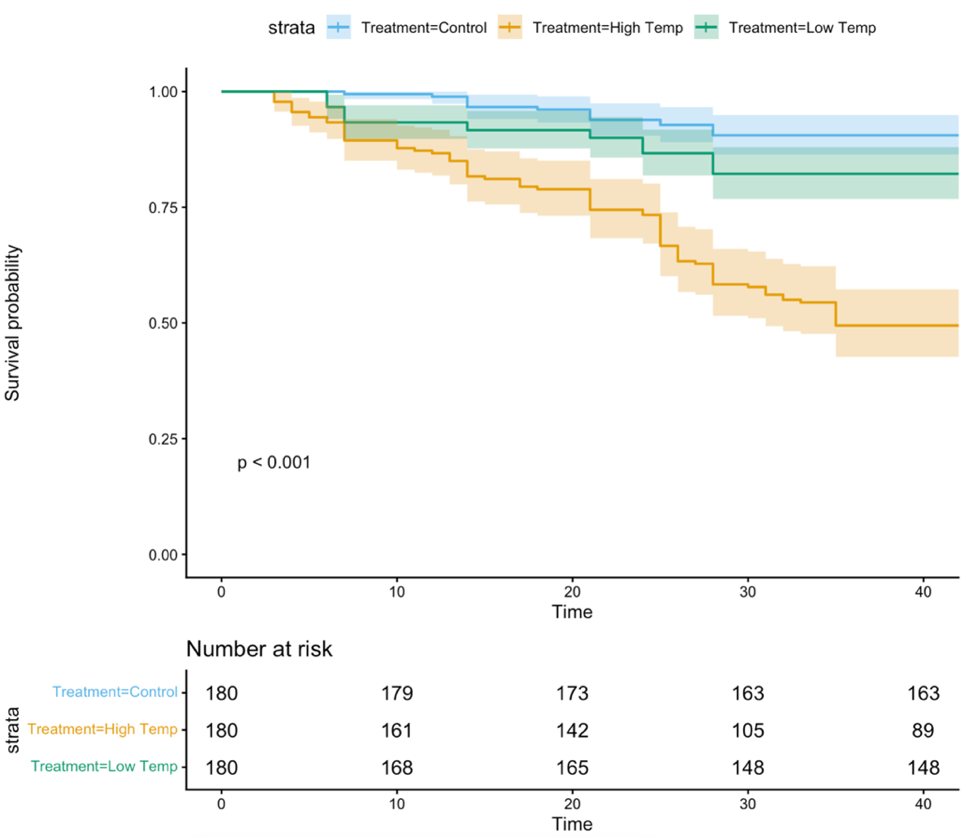


**Figure A1: The survival analysis of the controlled temperature experiment**

Supplement: Supplementary file 6 — Supplementary Material 6. Result from general mixed function (mean temperature). [file 40249_2026_1475_MOESM6_ESM.docx]
