# Supplementary material for: Impact of climate warming on Oncomelania hupensis in China: multi-scale evidence
Source: Infect Dis Poverty. 2026 Jul 3;15:76. doi: 10.1186/s40249-026-01475-0 (PMC13330383; doi:10.1186/s40249-026-01475-0)
Supplement: Supplementary file 7 — Supplementary Material 7. Results from Segmented Linear Models. [file 40249_2026_1475_MOESM7_ESM.docx]

**Table A1. Results from the Cox regression without temperature* body size interactions**

|  | **Coef** | ***χ^2^*** | ***p*** |
| --- | --- | --- | --- |
| High Temp vs Control | 1.79430 | 66.72 | 3.1e-16*** |
| Low Temp vs Control | -0.2756 | 0.81 | 0.371 |
| Body size | -0.53588 | 39.13 | 3.97e-10*** |
| Frailty (Tray) Variance=5.0e-7, *p*=0.92 | | | |
| Likelihood ratio test: χ² = 123.8, *p* < 0.001 | | | |

**Table A2. Results from Cox regression with temperature* body size interactions**

|  | **Coef** | ***χ^2^*** | ***p*** |
| --- | --- | --- | --- |
| Treatment: High Temp | 1.68882 | 51.08 | 8.9e-13*** |
| Treatment: Low Temp | -0.39992 | 1.55 | 0.2145 |
| Body size (before experiment) | -0.2913 | 3.15 | 7.6e-3** |
| Treatment: High Temp: Body size (before experiment) | -0.28704 | 3.06 | 0.08 |
| Treatment: Low Temp: Body size (before experiment) | 0.08023 | 0.02 | 0.87 |
| Frailty (Tray) Variance= 0.0092, *p*= 0.13 | | | |
| Likelihood ratio test: χ² = 127.4, *p* < 0.001 |  |  |  |
