# Supplementary material for: Impact of climate warming on Oncomelania hupensis in China: multi-scale evidence
Source: Infect Dis Poverty. 2026 Jul 3;15:76. doi: 10.1186/s40249-026-01475-0 (PMC13330383; doi:10.1186/s40249-026-01475-0)
Supplement: Supplementary file 8 — Supplementary Material 8. Distribution of exposure midpoint. [file 40249_2026_1475_MOESM8_ESM.docx]

**Table A1 Comparison of Body weights before and after the experiment**

| **Treatment** | **Before weight (95% *CI*)** | **After weight (95% *CI*)** | ***p*** |
| --- | --- | --- | --- |
| High temp | 33  (27.2, 38.8) | 25.5  (21.2, 29.8) | 0.0252* |
| Control | 28  (20.5, 35.5) | 31  (24, 38) | 0.477 |
| Low temp | 34  (29, 39) | 26.5  (22, 31) | 0.0323* |
