# Supplementary material for: Impact of climate warming on Oncomelania hupensis in China: multi-scale evidence
Source: Infect Dis Poverty. 2026 Jul 3;15:76. doi: 10.1186/s40249-026-01475-0 (PMC13330383; doi:10.1186/s40249-026-01475-0)
Supplement: Supplementary file 9 — Supplementary Material 9. Correlation between land use cover and Oncomelania hupensis density. [file 40249_2026_1475_MOESM9_ESM.docx]

1. *Results from the time-window model.*


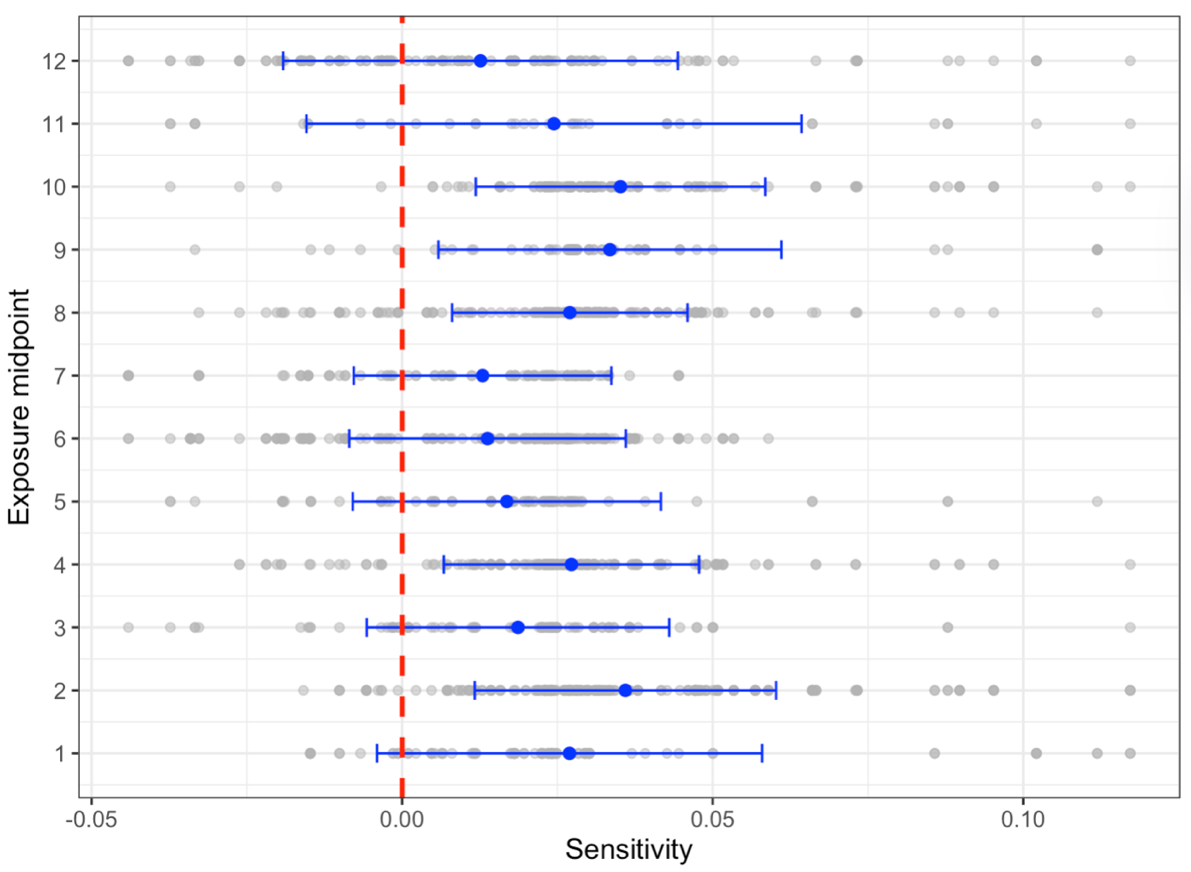


**Figure A1: Relationship between the midpoint and sensitivity to minimum temperature**


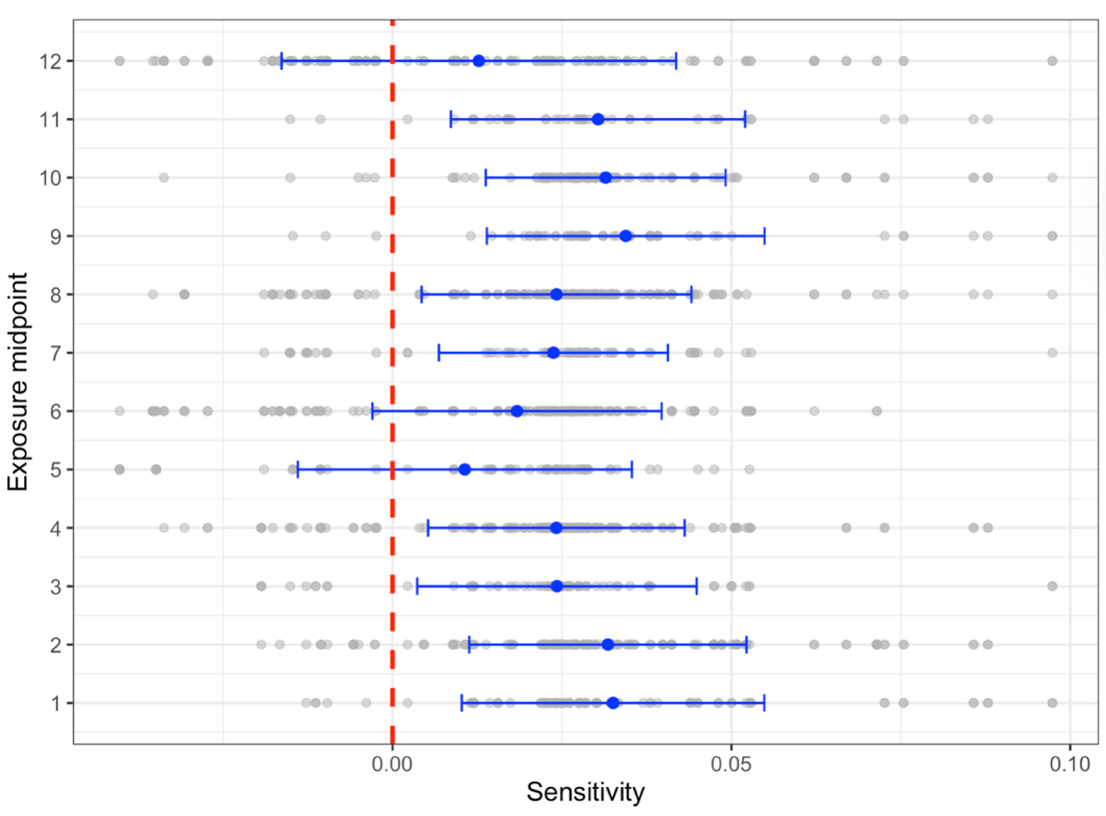


**Figure A2: Relationship between the midpoint and sensitivity to maximum temperature**
