# Supplementary material for: Impact of climate warming on Oncomelania hupensis in China: multi-scale evidence
Source: Infect Dis Poverty. 2026 Jul 3;15:76. doi: 10.1186/s40249-026-01475-0 (PMC13330383; doi:10.1186/s40249-026-01475-0)
Supplement: Supplementary file 10 — Supplementary Material 10. Result from general mixed function (minimum temperature) [file 40249_2026_1475_MOESM10_ESM.docx]

**Table A1: Result from general mixed function (maximum temperature)**

|  | **Estimate**  **(95% *CI*)** | ***t*** | ***p*** |
| --- | --- | --- | --- |
| **Temperature sensitivity** | | | |
| (Intercept) | -6.629e-02  (-1.021625e-01, -2.846555e-02) | -3.629 | 0.000334 *** |
| Latitude | 2.051e-03  (1.675388e-03, 2.414539e-03) | 10.908 | < 2e-16 *** |
| Longitude | 1.222e-04  (-1.807686e-04, 4.090361e-04) | 0.833 | 0.405475 |
| Dem | 1.574e-05  (1.411821e-05, 1.732204e-05) | 19.276 | < 2e-16 *** |
| Standardized temperature anomaly | 1.209e-01  (6.636397e-02, 1.748913e-01) | 4.366 | 1.27e-05 *** |
| Forest | -3.020e-03  (-3.942892e-03, -2.101130e-03) | -6.426 | 1.35e-10 *** |
| Grass | -3.356e-03  (-5.360047e-03, -1.336566e-03) | -3.270 | 0.001077 ** |
| Crop | -4.427e-03  (-5.195208e-03, -3.659572e-03) | -11.295 | < 2e-16 *** |
| Waterbody | -1.637e-03  (-2.921895e-03, -3.507889e-04) | -2.494 | 0.012624 * |
| *υ*: 1.663229; *ρ*: 13.823774 | | | |
| **Exposure duration** | | | |
| (Intercept) | 1.023e+01  (5.1337716847, 15.3404298055) | 3.898 | 0.000115 *** |
| Latitude | 2.465e-01  (0.1938859845, 0.2989479424) | 9.177 | < 2e-16 *** |
| Longitude | -8.284e-02  (-0.1237994474, -0.0418495511) | -3.933 | 9.48e-05 *** |
| Dem | -5.553e-04  (-0.0007844853, -0.0003279624) | -4.764 | 1.92e-06 *** |
| Standardized temperature anomaly | -3.978  (-4.7543477519, -3.2062335382) | -10.072 | < 2e-16 *** |
| Forest | -3.237e-01  (-0.4545020208, -0.1915266892) | -4.825 | 1.41e-06 *** |
| Grass | 9.971e-01  (0.7103880550, 1.2842698171) | 6.809 | 1.02e-11 *** |
| Crop | -7.961e-01  (-0.9052438019, -0.6859761627) | -14.233 | < 2e-16 *** |
| Waterbody | -9.544e-01  (-1.1374213377, -0.7705495735) | -10.196 | < 2e-16 *** |
| *υ*: 0.1130725; *ρ*: 1.6622221 | | | |
| **Exposure timing (midpoint)** | | | |
| (Intercept) | -3.598e+01  (-4.233776e+01, -29.132282267) | -11.155 | < 2e-16 *** |
| Latitude | 3.971e-01  (3.326097e-01, 0.458751197) | 12.503 | < 2e-16 *** |
| Longitude | 3.510e-02  (-1.888136e-02, 0.085183180) | 1.373 | 0.16998 |
| Dem | -1.071e-04  (-3.758943e-04, 0.000161541) | -0.780 | 0.43519 |
| Standardized temperature anomaly | 6.560e-02  (-1.493812e-01, 0.2779885330) | 0.601 | 0.54756 |
| Forest | 1.417  (1.260909, 1.569808115) | 17.998 | < 2e-16 *** |
| Grass | -1.371  (-1.706806, -1.033940565) | -7.988 | 1.46e-15 *** |
| Crop | 2.058e-01  (7.613276e-02, 0.333677582) | 3.136 | 0.00172 ** |
| Waterbody | 9.025e-01  (6.869391e-01, 1.117060378) | 8.225 | < 2e-16 *** |
| *υ*: 0.1489416; *ρ*: 4.5004726 | | | |
