# Supplementary material for: Impact of climate warming on Oncomelania hupensis in China: multi-scale evidence
Source: Infect Dis Poverty. 2026 Jul 3;15:76. doi: 10.1186/s40249-026-01475-0 (PMC13330383; doi:10.1186/s40249-026-01475-0)
Supplement: Supplementary file 11 — Supplementary Material 11. Result from the general mixed model with interaction between land use and standardized temperature anomaly. [file 40249_2026_1475_MOESM11_ESM.docx]

**Table A1: Result from general mixed function (mean temperature)**

|  | **Estimate**  **(*95% CI*)** | ***t* value** | ***p*** |
| --- | --- | --- | --- |
| **Temperature sensitivity** | | | |
| (Intercept) | 2.618e-01  (2.189694e-01, 3.052942e-01) | 11.859 | < 2e-16 *** |
| Latitude | 6.602e-04  (2.139819e-04, 1.093732e-03) | 2.956 | 0.003124 ** |
| Longitude | -2.061e-03  (-2.401206e-03, -1.725498e-03) | -11.923 | < 2e-16 *** |
| Dem | -1.065e-05  (-1.231211e-05, -8.970355e-06) | -12.504 | < 2e-16 *** |
| Standardized temperature anomaly | -2.063e-02  (-2.729414e-02, -1.396743e-02) | -6.067 | 1.33e-09 *** |
| Forest | -1.852e-03  (-2.950388e-03, -7.598015e-04) | -3.314 | 0.000921 *** |
| Grass | -1.006e-02  (-1.271917e-02, -7.409621e-03) | -7.429 | 1.15e-13 *** |
| Crop | -4.496e-03  (-5.408723e-03, -3.590311e-03) | -9.693 | < 2e-16 *** |
| Waterbody | 5.937e-03  (4.326149e-03, 7.549600e-03) | 7.218 | 5.51e-13 *** |
| *υ*: 0.01846871; *ρ*:0.04893386 | | | |
| **Exposure duration** |  |  |  |
| (Intercept) | -1.507e+01  (-1.954442e+01, -1.041748e+01) | -6.571 | 9.85e-11 *** |
| Latitude | -7.855e-02  (-1.221965e-01, -3.579211e-02) | -3.574 | 0.000353 *** |
| Longitude | 2.277e-01  (1.920658e-01, 2.615614e-01) | 13.093 | < 2e-16 *** |
| Dem | -4.107e-04  (-5.750073e-04, -2.476586e-04) | -4.919 | 8.80e-07 *** |
| Standardized temperature anomaly | -4.849  (-5.502712, -4.198694) | -14.576 | < 2e-16 *** |
| Forest | -3.929e-02  (-1.465402e-01, 6.765161e-02) | -0.719 | 0.472218 |
| Grass | -7.665e-01  (-1.025571, -5.064081e-01) | -5.787 | 7.29e-09 *** |
| Crop | -9.582e-01  (-1.047402, -8.695332e-01) | -21.118 | < 2e-16 *** |
| Waterbody | -3.754e-01  (-5.329752e-01, -2.178672e-01) | -4.669 | 3.05e-06 *** |
| *υ*: 0.04873447; *ρ*:1.64427853 | | | |
| **Exposure timing (midpoint)** | | | |
| (Intercept) | -1.535e+01  (-2.164118e+01, -9.2440232542) | -4.892 | 1.84e-06 *** |
| Latitude | -1.939e-01  (-2.583361e-01, -0.1271178345) | -5.822 | 6.27e-09 *** |
| Longitude | 3.259e-02  (-1.560231e-02, 0.0821665292) | 1.312 | 0.190 |
| Dem | 1.098e-04  (-1.395648e-04, 0.0003614594) | 0.858 | 0.391 |
| Standardized temperature anomaly | 2.165e+01  (2.065394e+01, 22.6626107497) | 42.252 | < 2e-16 *** |
| Forest | 1.316  (1.150628, 1.4808647748) | 15.610 | < 2e-16 *** |
| Grass | 1.047e-01  (-2.961933e-01, 0.5048006847) | 0.512 | 0.608 |
| Crop | 7.151e-01  (5.782198e-01, 0.8521483799) | 10.228 | < 2e-16 *** |
| Waterbody | 5.156e-02  (-2.955600e-01, 0.1908832861) | -0.415 | 0.678 |
| *υ*: 0.05792699; *ρ*: 0.26245594 | | | |
| **Density growth rate** | | | |
| (Intercept) | -2.961e-01  (-1.644474, 1.126129247) | -0.435 | 0.6643 |
| Latitude | 6.430e-03  (-8.329120e-03, 0.020596540) | 0.877 | 0.3808 |
| Longitude | -2.883e-03  (-1.408607e-02, 0.007743641) | -0.534 | 0.5942 |
| Dem | 6.999e-05  (1.350756e-05, 0.000124641) | 2.478 | 0.0132 * |
| Standardized temperature anomaly | 3.174e-01  (9.257698e-02, 0.537366962) | 2.801 | 0.0051 ** |
| Forest | -3.847e-02  (-7.493171e-02, -0.001917442) | -2.064 | 0.0390 * |
| Grass | -7.195e-02  (-1.602740e-01, 0.016879322) | -1.592 | 0.1114 |
| Crop | -8.416e-02  (-1.144193e-01, -0.053868142) | -5.445 | 5.25e-08 *** |
| Waterbody | 1.637e-01  (1.101217e-01, 0.217696482) | 5.966 | 2.48e-09 *** |
| Density | 2.801e-01  [0.1407801543, 0.4146420081] | 11.618 | < 2e-16 *** |
| *υ*: 1.234732; *ρ*: 1.6147945 | | | |
| **Density** | | | |
| (Intercept) | 2.958  (2.3773317999, 3.5369868321) | 9.932 | < 2e-16 *** |
| Latitude | 1.377e-02  (0.0079580729, 0.0197312417) | 4.599 | 4.31e-06 *** |
| Longitude | -2.996e-02  (-0.0346089333, -0.0253351225) | -12.598 | < 2e-16 *** |
| Dem | -2.140e-04  (-0.0002395623, -0.0001887264) | -16.503 | < 2e-16 *** |
| Standardized temperature anomaly | 2.800e-01  (0.1941455715, 0.3660228736) | 6.384 | 1.78e-10 *** |
| Forest | 9.448e-02  (0.0799319132, 0.1091327621) | 12.683 | < 2e-16 *** |
| Grass | 4.223e-03  (-0.0275869653, 0.0361249242) | 0.260 | 0.795 |
| Crop | 3.653e-02  (0.0243943530, 0.0487441546) | 5.880 | 4.18e-09 *** |
| Waterbody | 4.253e-02  (0.0221739773, 0.0628985359) | 4.093 | 4.28e-05 *** |
| *υ*: 0.02639659; *ρ*: 0.40946288 | | | |
