# Supplementary material for: Impact of climate warming on Oncomelania hupensis in China: multi-scale evidence
Source: Infect Dis Poverty. 2026 Jul 3;15:76. doi: 10.1186/s40249-026-01475-0 (PMC13330383; doi:10.1186/s40249-026-01475-0)
Supplement: Supplementary file 12 — Supplementary Material 12. Model diagnosis results of general linear mixed-effect models. [file 40249_2026_1475_MOESM12_ESM.docx]

*Results from segmented linear models*

We further used segmented linear models to identify altitudinal breakpoints in temperature-sensitivity. Mean temperature-sensitivity increased rapidly with altitude below 13.700 m (95% *CI*: 7.000, 14.000) but showed a more gradual increase at higher altitudes. In contrast, maximum temperature-sensitivity increased slightly with altitude below 1663.000 m (95% *CI*: 1493.900, 1775.000), whereas it increased rapidly above this threshold.

**Table A1: Model Evaluations of Segmented Linear Models with 0, 1, and 2 Breakpoint(s)**

|  | **AIC** | **BIC** |
| --- | --- | --- |
| **Minimum temperature sensitivity** | | |
| 0 break | -8210.610 | -8194.163 |
| 1 break | -8231.620 | -8204.209 |
| 2 breaks | -8253.115 | -8214.740 |
| **Mean temperature sensitivity** | | |
| 0 break | -7560.547 | -7544.260 |
| 1 break | -7607.565 | -7580.421 |
| 2 breaks | -7627.236 | -7589.234 |
| **Maximum temperature sensitivity** | | |
| 0 break | -8327.579 | -8311.255 |
| 1 break | -8375.124 | -8347.917 |
| 2 breaks | -8455.096 | -8417.007 |

**Table A2. Breakpoints of Segmented Linear Models and their Bootstrap 95% Confidence Intervals**

|  | **One break** | **Two breaks** |
| --- | --- | --- |
| **Minimum temperature sensitivity** | | |
| Success rate | 1000/1000 | 981/1000 |
| Breakpoint 1 95% *CI* | 1796 [127.8541, 1850.0243] | 162.5 [7.00, 1615.25] |
| Breakpoint 2 95% *CI* | - | 1849.8 [142.95, 1942.64] |
| **Mean temperature sensitivity** | | |
| Success rate | 1000/1000 | 966/1000 |
| Breakpoint 1 95% *CI* | **13.7 [7.00000 14.00001]** | 14.19 [12.87, 1898.00] |
| Breakpoint 2 95% *CI* | - | 22.00 [20.11, 1949.00] |
| **Maximum temperature sensitivity** | | |
| Success rate | 1000/1000 | 975/1000 |
| Breakpoint 1 95% *CI* | **1663 [1493.883 1774.992]** | 1309 [25.00, 1873.64] |
| Breakpoint 2 95% *CI* | - | 1383 [1321.00, 1948.99] |

Criteria for identifying breakpoint(s) were as follows:

1. The segmented model exhibited a lower AIC (and BIC) than the corresponding linear model without breakpoints.
2. Bootstrap 95% confidence intervals of the estimated breakpoint(s) were relatively narrow and showed no substantial overlap.
3. We choose the model with fewer breaks when two or more models have close AICs and BICs.

**Table A3: Results from Segmented Linear Models**

|  | **Estimates** |
| --- | --- |
| **Mean temperature sensitivity (Estimated Breakpoints: 13.700)** | |
| (Intercept) | -0.010577 |
| DEM (before break) | 0.2289 |
| DEM (after break) | 0.002282 |
| **Maximum temperature sensitivity (Estimated Breakpoints: 1663)** | |
| (Intercept) | 2.044e-02 |
| DEM (before break) | 4.532e-06 |
| DEM (after break) | 3.742e-05 |
