# Supplementary material for: Impact of climate warming on Oncomelania hupensis in China: multi-scale evidence
Source: Infect Dis Poverty. 2026 Jul 3;15:76. doi: 10.1186/s40249-026-01475-0 (PMC13330383; doi:10.1186/s40249-026-01475-0)
Supplement: Supplementary file 13 — Supplementary Material 13. The population benefits from global warming under SSP1-2.6, SSP2-4.5, and SSP5-8.5. [file 40249_2026_1475_MOESM13_ESM.docx]

**Table A1. Distribution of exposure midpoint (minimum temperature)**

| **Land use** | **Midpoint (the minimum temperature)** | **n** |
| --- | --- | --- |
| Impervious area | February | 50 |
| Forest | August | 76 |
| Grass | February | 10 |
| Crop | June | 132 |
| Waterbody | April | 28 |

**Table A2. Distribution of exposure midpoint (mean temperature)**

| **Land use** | **Midpoint (mean temperature)** | **n** |
| --- | --- | --- |
| Impervious are | April | 54 |
| Forest | June | 59 |
| Grass | February | 10 |
| Crop | August | 120 |
| Waterbody | April | 33 |

**Table A3. Distribution of exposure midpoint (maximum temperature)**

| **Land use** | **Midpoint (the maximum temperature)** | **n** |
| --- | --- | --- |
| Impervious area | February | 56 |
| Forest | August | 68 |
| Grass | August | 12 |
| Crop | June | 121 |
| Waterbody | June | 33 |
