# Supplementary material for: Impact of climate warming on Oncomelania hupensis in China: multi-scale evidence
Source: Infect Dis Poverty. 2026 Jul 3;15:76. doi: 10.1186/s40249-026-01475-0 (PMC13330383; doi:10.1186/s40249-026-01475-0)
Supplement: Supplementary file 14 — Supplementary Material 14. Distribution of population benefiting from climate warming across the land-uses under SSP1-2.6, SSP2-4.5, and SSP5-8.5. [file 40249_2026_1475_MOESM14_ESM.docx]

**Table A1. Correlation between land use cover and *Oncomelania hupensis* density**

| **Land use** | **Estimate** | ***t*** | ***p*** |
| --- | --- | --- | --- |
| **3km radius** | | | |
| (Intercept) | 0.1168174  (-0.0277326, 0.2613674) | 1.58397 | 0.128886 |
| Crop | 0.2427284  (0.221286, 0.2641708) | 3.105 | 0.002265 ** |
| Forest | -0.1056565  (-0.4057325, 0.1944195) | -1.3185 | 0.1893 |
| Grass | -0.08536784  (-0.4640398, 0.2933042) | -1.0633 | 0.2893 |
| Impervious area | -0.1039812  (-0.2147212, 0.0067588) | -1.2974 | 0.1964 |
| Waterbody | -0.0558447  (-0.1665847, 0.0548953) | -0.6941 | 0.4887 |
| *υ*: 0.1876553; *ρ*: 1.7040460 | | | |
| **1km radius** | | | |
| (Intercept) | -1.91992  (-5.16123, 1.32139) | -0.5923 | 0.5602871 |
| Crop | 0.1091726  (0.0767426, 0.1416026) | 9.4101 | < 2.2e-16 *** |
| Forest | -0.02583763  (-0.04856763, -0.00310763) | -2.2145 | 0.02683 * |
| Grass | -0.0201508  (-0.0526708, 0.0123692) | -1.7269 | 0.08423 |
| Impervious area | -0.01279869  (-0.04531869, 0.01972131) | -1.0967 | 0.2728 |
| Waterbody | -0.04790483  (-0.08042483, -0.01538483) | -4.1092 | 4.014e-05 *** |
| *υ*: 1.048549; *ρ*: 3.308534 | | | |
| **5km radius** | | | |
| (Intercept) | -3.07009  (-7.16953,1.02935) | -0.7489 | 0.4626351 |
| Crop | 0.1811342  (0.1401242, 0.2221442) | 2.3224 | 0.02148 * |
| Forest | -0.101019  (-0.514619, 0.312581) | -1.2804 | 0.2023 |
| Grass | -0.09148429  (-0.1876013, 0.00463271) | -1.1584 | 0.2484 |
| Impervious area | 0.002520139  (-0.001588861, 0.006629139) | 0.031778 | 0.9747 |
| Waterbody | -0.04036441  (-0.08155441, 0.00082559) | -0.50939 | 0.6112 |
| *υ*: 2.923344; *ρ*: 5.667293 | | | |
