# Supplementary material for: Impact of climate warming on Oncomelania hupensis in China: multi-scale evidence
Source: Infect Dis Poverty. 2026 Jul 3;15:76. doi: 10.1186/s40249-026-01475-0 (PMC13330383; doi:10.1186/s40249-026-01475-0)
Supplement: Supplementary file 15 — Supplementary Material 15. Predicted density across land uses under SSP1-2.6, SSP2-4.5, SSP5-8.5. [file 40249_2026_1475_MOESM15_ESM.docx]

**Table A1: Result from general mixed function (minimum temperature)**

|  | **Estimate**  **(95% *CI*)** | ***t* value** | ***p*** |
| --- | --- | --- | --- |
| **Temperature sensitivity** | | | |
| (Intercept) | 6.176e-01  (5.690363e-01, 6.656179e-01) | 25.055 | < 2e-16 *** |
| Latitude | -2.260e-03  (-2.675823e-03, -1.837959e-03) | -10.583 | < 2e-16 *** |
| Longitude | -4.525e-03  (-4.867199e-03, -4.170948e-03) | -25.673 | < 2e-16 *** |
| Dem | -3.919e-06  (-5.724292e-06, -2.103161e-06) | -4.243 | 2.22e-05 *** |
| Standardized temperature anomaly | -9.567e-03  (-1.568769e-02, -3.427013e-03) | -3.058 | 0.00223 ** |
| Forest | 1.377e-03  (3.346612e-04, 2.424352e-03) | 2.583 | 0.00979 ** |
| Grass | 1.927e-02  (1.700147e-02, 2.153514e-02) | 16.661 | < 2e-16 *** |
| Crop | 2.670e-03  (1.799158e-03, 3.543994e-03) | 5.999 | 2.03e-09 *** |
| Waterbody | 4.440e-03  (2.946987e-03, 5.934906e-03) | 5.824 | 5.85e-09 *** |
| *υ*: 0.005241203; *ρ*: 3.523292538. | | | |
| **Exposure duration** | | | |
| (Intercept) | -1.048e+01  (-15.645722052, -5.0007331855) | -3.972 | 7.74e-05 *** |
| Latitude | -4.454e-03  (-0.055376272, 0.0449343965) | -0.175 | 0.861 |
| Longitude | 1.483e-01  (0.105260202, 0.1886139938) | 7.182 | 9.35e-13 *** |
| Dem | -6.135e-04  (-0.000830665, -0.0003974228) | -5.549 | 2.93e-08 *** |
| Standardized temperature anomaly | -3.250  (-3.986075391, -2.5152037938) | -8.659 | < 2e-16 *** |
| Forest | 1.347  (1.220472592, 1.4714055976) | 21.073 | < 2e-16 *** |
| Grass | -1.542e-01  (-0.425719362, 0.1183365484) | -1.111 | 0.267 |
| Crop | 1.042  (0.936477499, 1.1459611364) | 19.515 | < 2e-16 *** |
| Waterbody | 1.058  (0.877836647, 1.2365078735) | 11.562 | < 2e-16 *** |
| *υ*: 0.27040267; *ρ*: 0.03611611 | | | |
| **Exposure timing (midpoint)** | | | |
| (Intercept) | -7.732e+01  (-83.625597660, -70.735597433) | -23.990 | < 2e-16 *** |
| Latitude | 1.497e-01  (0.090034379, 0.208076230) | 4.987 | 6.21e-07 *** |
| Longitude | 4.507e-01  (0.399753448, 0.498696471) | 18.290 | < 2e-16 *** |
| Dem | 1.397e-03  (0.001140761, 0.001650743) | 10.741 | < 2e-16 *** |
| Standardized temperature anomaly | 2.563e+01  (24.759836774, 26.487457291) | 58.139 | < 2e-16 *** |
| Forest | 8.307e-02  (-0.064925354, 0.229611825) | 1.106 | 0.269 |
| Grass | 9.140e-01  (0.595261290, 1.234159852) | 5.608 | 2.08e-08 *** |
| Crop | -3.050e-01  (-0.428379440, -0.182491321) | -4.863 | 1.17e-06 *** |
| Waterbody | -1.448  (-1.658854346, -1.237784226) | -13.478 | < 2e-16 *** |
| *υ*: 0.1320381; *ρ*: 2.5041988 | | | |
