# Supplementary material for: Impact of climate warming on Oncomelania hupensis in China: multi-scale evidence
Source: Infect Dis Poverty. 2026 Jul 3;15:76. doi: 10.1186/s40249-026-01475-0 (PMC13330383; doi:10.1186/s40249-026-01475-0)
Supplement: Supplementary file 16 — Supplementary Material 16. Predicted growth rate across land uses in GCMs. [file 40249_2026_1475_MOESM16_ESM.docx]

**Table A1. Interaction between land use and standardized temperature anomaly** (result from the general mixed model calculated using mean temperatures)

|  | **Estimate**  **(95% *CI*)** | ***t*** | ***p*** |
| --- | --- | --- | --- |
| (Intercept) | 7.123e-01  (0.1540256867, 1.267485e+00) | 2.502 | 0.01247 * |
| Latitude | 5.839e-03  (0.0012258012, 1.044412e-02) | 2.486 | 0.01293 * |
| Longitude | -5.849e-03  (-0.0100161875, -1.647118e-03) | -2.739 | 0.00618 ** |
| Dem | -7.378e-05  (-0.0000943088, -5.334056e-05) | -7.071 | 1.55e-12 *** |
| Standardized temperature anomaly (STA) | -1.898e-02  (-0.2030562625, 1.650519e-01) | -0.202 | 0.83971 |
| Forest | -7.229e-02  (-0.3388117532, 1.914222e-01) | -0.535 | 0.59257 |
| Grass | 6.068e-01  (-0.5377407086, 1.753544e+00) | 1.039 | 0.29890 |
| Crop | 1.819e-01  (-0.0365361528, 4.005092e-01) | 1.633 | 0.10252 |
| Waterbody | -5.977e-01  (-1.0578750961, -1.375671e-01) | -2.547 | 0.01086 * |
| Forest: STA | 9.684e-02  (-0.1445979312, 3.410876e-01) | 0.783 | 0.43390 |
| Grass: STA | -5.644e-01  (-1.6512388077, 5.199615e-01) | -1.020 | 0.30789 |
| Crop: STA | -1.529e-01  (-0.3527492639, 4.684717e-02) | -1.501 | 0.13340 |
| Water: STA | 5.208e-01  (0.0984310935, 9.432104e-01) | 2.418 |  |
